# Joint super-resolution and synthesis of 1 mm isotropic MP-RAGE volumes from clinical MRI exams with scans of different orientation, resolution and contrast

**DOI:** 10.1016/j.neuroimage.2021.118206

**Published:** 2021-08-15

**Authors:** Juan Eugenio Iglesias, Benjamin Billot, Yaël Balbastre, Azadeh Tabari, John Conklin, R. Gilberto González, Daniel C. Alexander, Polina Golland, Brian L. Edlow, Bruce Fischl, for the Alzheimer’s Disease Neuroimaging Initiative

**Affiliations:** Data used in preparation of this article were obtained from the Alzheimers Disease Neuroimaging Initiative (ADNI) database (adni.loni.usc.edu). As such, the investigators within the ADNI contributed to the design and implementation of ADNI and/or provided data but did not participate in analysis or writing of this report. A complete listing of ADNI investigators can be found at: http://adni.loni.usc.edu/wp-content/uploads/how_to_apply/ADNI_Acknowledgement_List.pdf; a Centre for Medical Image Computing, Department of Medical Physics and Biomedical Engineering, University College London, UK; b Athinoula A. Martinos Center for Biomedical Imaging, Massachusetts General Hospital and Harvard Medical School, Boston, USA; c Computer Science and Artificial Intelligence Laboratory, Massachusetts Institute of Technology, Boston, USA; d Department of Radiology, Massachusetts General Hospital, Boston, USA; e Neuroradiology Division, Massachusetts General Hospital, Boston, USA; f Center for Neurotechnology and Neurorecovery, Massachusetts General Hospital, Boston, USA

**Keywords:** Super-resolution, Clinical scans, Convolutional neural network, Public software

## Abstract

Most existing algorithms for automatic 3D morphometry of human brain MRI scans are designed for data with near-isotropic voxels at approximately 1 mm resolution, and frequently have contrast constraints as well-typically requiring T1-weighted images (e.g., MP-RAGE scans). This limitation prevents the analysis of millions of MRI scans acquired with large inter-slice spacing in clinical settings every year. In turn, the inability to quantitatively analyze these scans hinders the adoption of quantitative neuro imaging in healthcare, and also precludes research studies that could attain huge sample sizes and hence greatly improve our understanding of the human brain. Recent advances in convolutional neural networks (CNNs) are producing outstanding results in super-resolution and contrast synthesis of MRI. However, these approaches are very sensitive to the specific combination of contrast, resolution and orientation of the input images, and thus do not generalize to diverse clinical acquisition protocols – even within sites. In this article, we present *SynthSR*, a method to train a CNN that receives one or more scans with spaced slices, acquired with different contrast, resolution and orientation, and produces an isotropic scan of canonical contrast (typically a 1 mm MP-RAGE). The presented method does not require any preprocessing, beyond rigid coregistration of the input scans. Crucially, *SynthSR* trains on synthetic input images generated from 3D segmentations, and can thus be used to train CNNs for *any* combination of contrasts, resolutions and orientations without high-resolution real images of the input contrasts. We test the images generated with *SynthSR* in an array of common downstream analyses, and show that they can be reliably used for subcortical segmentation and volumetry, image registration (e.g., for tensor-based morphometry), and, if some image quality requirements are met, even cortical thickness morphometry. The source code is publicly available at https://github.com/BBillot/SynthSR.

## Highlights

• SynthSR turns clinical scans of different resolution and contrast into 1 mm MPRAGEs.
• It relies on a CNN trained on fake images synthesized on the fly at every minibatch.
• It can be retrained for any combination of resolutions / contrasts without new data.
• It enables segmentation, registration, etc with existing software (e.g. FreeSurfer) Code is open source.

## Graphical abstract

**Figure:**
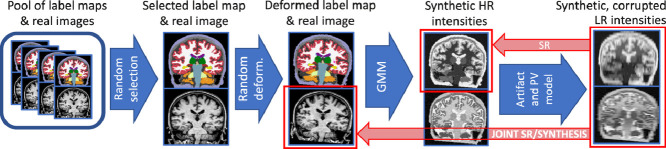

## 1 Introduction

### 1.1 Motivation

Magnetic resonance imaging (MRI) has revolutionized research on the human brain, by enabling *in vivo* noninvasive neuroimaging with exquisite and tunable soft-tissue contrast. Quantitative and reproducible analysis of brain scans requires automated algorithms that analyze brain morphometry in 3D, and thus best operate on data with isotropic voxels. Most existing human MRI image analysis methods only produce accurate results when they operate on near-isotropic acquisitions that are commonplace in research, and their performance often drops quickly as voxel size and anisotropy increase. Examples of such methods include most of the tools in the major packages that arguably drive the field (FreeSurfer, Fischl, 2012; FSL, Jenkinson et al., 2012; SPM, Ashburner, 2012; or AFNI, Cox, 1996), e.g., for segmentation (Ashburner, Friston, 2005, Dale, Fischl, Sereno, 1999, Fischl, Salat, Busa, Albert, Dieterich, Haselgrove, Van Der Kouwe, Killiany, Kennedy, Klaveness, et al., 2002, Patenaude, Smith, Kennedy, Jenkinson, 2011) or registration (Andersson, Jenkinson, Smith, et al., 2007, Ashburner, 2007, Cox, Jesmanowicz, 1999, Greve, Fischl, 2009, Jenkinson, Bannister, Brady, Smith, 2002) of brain MRI scans. Many other popular tools outside these packages also exhibit decreased accuracy when processing anisotropic scans, including registration packages like ANTs (Avants et al., 2008), Elastix (Klein et al., 2009) or NiftyReg (Modat et al., 2010), particularly in nonlinear mode; and modern segmentation methods based on convolutional neural networks (CNNs) and particularly the U-net architecture (Çiçek, Abdulkadir, Lienkamp, Brox, Ronneberger, 2016, Ronneberger, Fischer, Brox, 2015), such as DeepMedic (Kamnitsas et al., 2017), DeepNAT (Roy, Conjeti, Navab, Wachinger, Initiative, et al., 2019, Wachinger, Reuter, Klein, 2018), or VoxResNet (Chen et al., 2018a).

Moreover, many of these tools require specific sequences and types of MR contrast to differentiate gray and white matter, such as the ubiquitous MP-RAGE sequence (Mugler and Brookeman, 1990) and its variants (van der Kouwe, Benner, Salat, Fischl, 2008, Marques, Kober, Krueger, van der Zwaag, Van de Moortele, Gruetter, 2010). Focusing on a specific MR contrast enables algorithms to be more accurate by learning prior distributions of intensities from labeled training data, but also limits their ability to analyze images with contrasts different from that of the training dataset. Most segmentation methods, with the notable exception of Bayesian algorithms with unsupervised likelihood (Ashburner, Friston, 2005, Van Leemput, Maes, Vandermeulen, Suetens, 1999), have this MRI contrast requirement, and deviations from the expected intensity profiles (“domain shift”, even within T1-weighted MRI) lead to decreased performance, even with intensity standardization techniques (Han et al., 2006). The loss of accuracy due to domain shift is particularly large for CNNs, which are fragile against changes in MRI contrast, resolution, or orientation (see, e.g., Billot, Greve, Van Leemput, Fischl, Iglesias, Dalca, 2020, Billot, Robinson, Dalca, Iglesias, 2020, Jog, Hoopes, Greve, Van Leemput, Fischl, 2019), unless equipped with sophisticated domain adaptation techniques (Wang and Deng, 2018). While classic registration algorithms are contrast agnostic, modern deep learning registration techniques (e.g., Balakrishnan, Zhao, Sabuncu, Guttag, Dalca, 2019, de Vos, Berendsen, Viergever, Sokooti, Staring, Išgum, 2019) also require images with MR contrast similar to that of the scans used in training.

However, MRI scans acquired in the clinic are typically quite different from those obtained as part of research studies. Rather than isotropic volumes, physicians have traditionally preferred a relatively sparse set of images of parallel planes, which reduces the time required for acquisition and visual inspection. Therefore, clinical MRI exams^2^ typically comprise several scans acquired with different orientations and (often 2D) pulse sequences, each of which consists of a relatively small set of slices (20–30) with large spacing in between (5–7 mm) and often high in-plane resolution (e.g., 0.5 mm). While morphometry of isotropic scans is also starting to be used in the clinic, quantitative imaging in clinical practice is still in its infancy, and the vast majority of existing clinical MRI scans – including decades of legacy data – are highly anisotropic, and thus cannot be reliably analyzed with existing tools.

The inability to analyze clinical data in 3D has deleterious consequences in the clinic and in research. In clinical practice, it precludes: quantitative evaluation of the status of a patient compared to the general population; precise measurement of longitudinal change; and reduction of variability in subjective evaluation due to the positioning of the slices. In research, this inability hinders the analysis of millions of brain scans that are currently stored in picture archiving and communication systems (PACS) around the world. Computing measurements from such clinical scans would thus enable research studies with statistical power levels that are currently unattainable, with large potential for improving our understanding of brain diseases.

### 1.2 Related work

There have been many attempts to bridge the gap between clinical and research scans in medical imaging, mostly based on super-resolution (SR) and synthesis techniques, many of which originated from the computer vision literature. SR seeks to obtain an enhanced, high-resolution (HR) image from an input consisting of one or multiple lower-resolution (LR) frames. Early SR was model-based and relied on multiple LR images of the same scene acquired with slight differences in camera positioning. More modern SR methods rely on machine learning (ML) techniques, which often use a dataset of matching LR-HR images to learn a mapping that enables recovery of HR from LR; training data are often obtained by blurring and subsampling HR images to obtain their LR counterparts. Classical ML methods have long been used to learn this mapping, including non-local patch techniques (Manjón et al., 2010), sparse representations (Rueda et al., 2013), low-rank methods (Shi et al., 2015), canonical correlation analysis (Bahrami et al., 2016), random forests (Alexander et al., 2017), or sparse coding (Huang et al., 2017).

These classical techniques have been superseded by deep CNNs, which have achieved very impressive results. Earlier methods relying on older and simpler architectures from the computer vision literature (e.g., Pham et al., 2017, based on the SRCNN architecture, Dong et al., 2015) already surpassed classical techniques by a large margin. Further improvements have been provided by the adoption of more recent developments in CNNs, such as densely connected networks (Chen et al., 2018c), adversarial networks (Chen et al., 2018b), residual connections (Chaudhari et al., 2018), uncertaintly modeling (Tanno et al., 2020), or progressive architectures (Lyu et al., 2020). Importantly, it has been shown that the SR images generated with such deep learning techniques can enhance downstream analyses. For example, Tian et al. (2020) showed improvements in cortical thickness estimation on super-resolved brain MRI scans, whereas Tanno et al. (2020) showed similar results in fiber tracking using super-resolved diffusion MRI. Delannoy et al. (2020) showed improved segmentation of neonatal brain MRI by solving SR and segmentation simultaneously. In MRI of organs other than the brain, SR has also been shown to produce improved results, for example in ventricular volume estimation in cardiac MRI (Masutani et al., 2020), or osteophyte detection in knee MRI (Chaudhari et al., 2020).

Meanwhile, MRI contrast synthesis techniques for brain imaging have followed a path parallel to SR. Early methods used classical ML techniques such as dictionary learning (Roy et al., 2011), patch matching (Iglesias et al., 2013), or random forests (Huynh et al., 2015). These methods have also been superseded by modern ML techniques based on CNNs, often equipped with adversarial losses (Goodfellow et al., 2014) to preserve finer, higher-frequency detail, as well as cycle consistency (Zhu et al., 2017) in order to enable synthesis with unpaired data (e.g., Chartsias, Joyce, Giuffrida, Tsaftaris, 2017, Dar, Yurt, Karacan, Erdem, Erdem, Çukur, 2019, Nie, Trullo, Lian, Wang, Petitjean, Ruan, Wang, Shen, 2018, Shin, Tenenholtz, Rogers, Schwarz, Senjem, Gunter, Andriole, Michalski, 2018, Xiang, Wang, Nie, Zhang, Jin, Qiao, Shen, 2018).

While the performance of CNNs in SR and synthesis of MRI is impressive, their adoption in clinical MRI analysis is hindered by the fact that they typically require paired training data, in order to yield good performance. While adversarial approaches can exploit HR images of the target contrast in training, they are normally used *in combination* with supervised voxel-level losses (Chen, Shi, Christodoulou, Xie, Zhou, Li, 2018, Ledig, Theis, Huszár, Caballero, Cunningham, Acosta, Aitken, Tejani, Totz, Wang, et al., 2017), since reduced accuracy is obtained when they are used in isolation (Song et al., 2020). This is an important limitation, as such required training data are most often not available – particularly since the combination of resolution, contrast and orientations acquired in brain MRI exams vary substantially across (and even within) sites. To tackle this problem, classical methods based on probabilistic models have been proposed. For example, Dalca et al. (2018) used collections of scans with spaced slices to build a generative model that they subsequently inverted to fill in the missing information between slices. Brudfors et al. (2018) also cast SR as an inverse problem, using multi-channel total variation as a prior; this approach has the advantage of not needing access to a collection of scans for training, so it can be immediately used for any new set of input contrasts. Jog et al. (2016) use Fourier Burst Accumulation (Delbracio and Sapiro, 2015) to super-resolve across slices using the high-resolution information existing within slices (i.e., in plane); as Brudfors et al. (2018), this technique can also applied to single images. Unfortunately, the performance of these classical approaches is lower than that of their CNN counterparts.

The closest works related to the technique proposed in this article are those by Huang et al. (2017); Zhao et al. (2020) and Du et al. (2020). Huang et al. present “WEENIE”, a weakly-supervised joint convolutional sparse coding method for joint SR and synthesis of brain MRI. WEENIE combines a small set of image pairs (LR of source domain, HR of target domain) with a larger set of unpaired scans, and uses convolutional sparse coding to learn a representation (a joint dictionary) where the similarity of the feature distributions of the paired and unpaired data is maximized. The main limitation of WEENIE is its need for paired data, even if in a small amount. Both Zhao et al. (2020) and Du et al. (2020) can be seen as deep learning versions of Jog et al. (2016), which rely on training a CNN with high-resolution slices (blurred along one of the two dimensions), and using this CNN to super-resolve the imaging volume across slices. While this technique does not require HR training data and can be applied to a single scan, it has two disadvantages compared with the method presented here. First, it is unable to combine the information from multiple scans from the same MRI exam, with different resolution and contrast. And second, integration of MR contrast synthesis into the method is not straightforward.

### 1.3 Contribution

Despite recent efforts to improve generalization ability across MR contrasts (see Billot et al., 2020a for an example in segmentation), the applicability deep learning SR and synthesis techniques to clinical MRI is often impractical due to substantial differences in MR acquisition protocols across sites – not only contrast, but also orientation and resolution. Even within a single site, it is common for brain MRI exams to comprise different sets of sequences – particularly when considering longitudinal data, since acquisition protocols are frequently updated and improved, and the same patients may be scanned on different platforms (possibly with different field strengths).

In this article we present *SynthSR*, a solution to this problem that uses synthetically generated images to train a CNN – an approach that we recently applied with success to contrast-agnostic and partial volume (PV) segmentation of brain MRI (Billot, Greve, Van Leemput, Fischl, Iglesias, Dalca, 2020, Billot, Robinson, Dalca, Iglesias, 2020). The synthetic data mimic multi-modal MRI scans with channels of different resolutions and contrasts, and include artifacts such as bias fields, registration errors, and resampling artifacts. Having full control over the generative process allows us to train CNNs for super-resolution, synthesis, or both, for any desired combination of MR contrasts, resolution, and orientation – without ever observing a real HR scan of the target contrast, thus enabling wide applicability.

To the best of our knowledge, *SynthSR* is the first deep learning technique that enables “reconstruction” of an isotropic scan of a reference MRI contrast from a set of scans with spaced slices, acquired with different resolutions and pulse sequences. We extensively validate the applicability of our approach with image similarity metrics (peak signal-to-noise ratio, structural similarity) and also by analyzing the performance of common neuroimaging tools on the reconstructed isotropic scans, including: segmentation for volumetry, registration for tensor-based morphometry, and cortical thickness.

The rest of this paper is organized as follows: Section 2 describes our proposed framework to generate synthetic images, and how it can be used to train CNNs for SR, synthesis, or both simultaneously. Section 3 presents three different experiments that evaluate our proposed method with synthetic and real data, and compare its performance with Bayesian approaches. Finally, Section 4 discusses the results and concludes the article with a consideration of future directions and applications of this technique.

## 2 Methods

### 2.1 Synthetic data generator

**Fig. 1:**
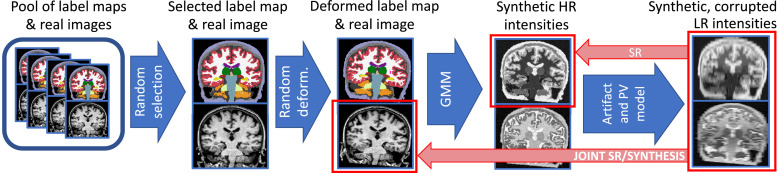
Overview of the synthetic data generator used by *SynthSR*. The blue arrows follow the generative model, which is used to sample random scans at every minibatch using a GPU implementation. The red arrows connect the inputs and regression targets used in training for SR or joint SR / synthesis. We emphasize that the real images are only required for joint SR / synthesis, and not SR alone. (For interpretation of the references to color in this figure legend, the reader is referred to the web version of this article.)

The cornerstone of *SynthSR* is a synthetic data generator that enables training CNNs for SR and synthesis using brain MRI scans of any resolution and contrast (Billot, Greve, Van Leemput, Fischl, Iglesias, Dalca, 2020, Billot, Robinson, Dalca, Iglesias, 2020). At every minibatch, this generator is used to randomly sample a series of synthetic images that are used to update the CNN weights via a regression loss. Crucially, this generator is implemented in the GPU, so it does not significantly slow down training. The flowchart of the generator is illustrated in Fig. 1; the different steps are described below.

#### 2.1.1 Sample selection

For training, we assume the availability of a pool of HR brain scans with the same MR contrast ${\left\{I_{n}\right\}}_{n=1,\ldots ,N}$, together with corresponding segmentations (“label maps”) of K classes ${\left\{L_{n}\right\}}_{n=1,\ldots ,N}$ corresponding to brain structures and extracerebral regions; these segmentations can be manual, automated, or a combination thereof. Importantly, the MR contrast of these volumes *defines* the reference contrast we will synthesize, so they would typically be 1 mm isotropic MP-RAGE scans; if one wishes to perform SR alone (i.e., without synthesis), these images are not required. At every minibatch, the generative process starts by randomly selecting an image-segmentation pair (I,L) from the pool using a uniform distribution:

$$\begin{array}{cc} n & ∼U(1,N),\\ I & \leftarrow I_{n},\\ L & \leftarrow L_{n}. \end{array}$$

#### 2.1.2 Spatial augmentation

The selected image and segmentation are augmented with a spatial transform T, which is the composition of a linear and nonlinear transform: $T=T_{lin}\circ T_{nonlin}$. The linear component is a combination of three rotations $\left(\theta_{x},\theta_{y},\theta_{z}\right)$, three scalings $\left(s_{x},s_{y},s_{z}\right)$ and three shearings $\left(\phi_{x},\phi_{y},\phi_{z}\right)$, all sampled from uniform distributions (the scalings are sampled in logarithmic domain):

(1) $$\begin{array}{cc} & \theta_{x}∼U\left(a_{rot},b_{rot}\right),\;\log s_{x}∼U\left(a_{sc},b_{sc}\right),\;\phi_{x}∼U\left(a_{sh},b_{sh}\right),\\ & \theta_{y}∼U\left(a_{rot},b_{rot}\right),\;\log s_{y}∼U\left(a_{sc},b_{sc}\right),\;\phi_{y}∼U\left(a_{sh},b_{sh}\right),\\ & \theta_{z}∼U\left(a_{rot},b_{rot}\right),\;\log s_{z}∼U\left(a_{sc},b_{sc}\right),\;\phi_{z}∼U\left(a_{sh},b_{sh}\right),\\ & T_{lin}=\text{Affine}\left(\theta_{x},\theta_{y},\theta_{z},s_{x},s_{y},s_{z},\phi_{x},\phi_{y},\phi_{z}\right), \end{array}$$

where $a_{rot},b_{rot},a_{sc},b_{sc},a_{sh},b_{sh}$ are the minimum and maximum values of the uniform distribution, and Affine(·) is an affine matrix consisting of the product of nine matrices: three scalings, three shearings, and three rotations about the x, y and z axis. We note that we do not include translation into the model, since it is not helpful in a dense prediction setup – as opposed to, e.g., image classification.

The nonlinear component is obtained by integrating a stationary velocity field (SVF), which yields a transform that is smooth and invertible almost everywhere (violations may happen due to discretization into voxels) – thus encouraging preservation of the topology of the segmentation (i.e., the brain anatomy). The transform is generated as follows. First, we generate a low dimensional volume with three channels (e.g., 10×10×10×3) by randomly sampling a zero-mean Gaussian distribution at each location independently. Second, we trilinearly upsample these three channels to the size of the image I in order to obtain a smooth volume with three channels, which we interpret as an SVF. Finally, we compute the Lie exponential via integration of the SVF with a scale-and-square approach (Arsigny et al., 2006) in order to obtain a nearly diffemorphic field that is smooth and invertible almost everywhere:

$$\begin{array}{cc} {\text{SVF}}^{\prime } & ∼N_{10\times 10\times 10\times 3}\left(0,\sigma_{T}^{2}\right),\\ \text{SVF} & =\text{Upsample}({\text{SVF}}^{\prime }),\\ T_{nonlin} & =\exp(\text{SVF}). \end{array}$$

where the variance $\sigma_{T}^{2}$ controls the smoothness of the field.

Finally, the composite deformation T is used to deform I and L into $I^{T}$ and $L^{T}$ using trilinear and nearest neighbor interpolation, respectively:

$$\begin{array}{cc} I^{T}=I\circ T & =I\circ (T_{lin}\circ T_{nonlin})\\ L^{T}=L\circ T & =L\circ (T_{lin}\circ T_{nonlin}) \end{array}$$

#### 2.1.3 Synthetic HR intensities

Given the deformed segmentation $L^{T}$, we subsequently generate HR intensities by sampling a Gaussian mixture model (GMM) at each location, conditioned on the labels. This GMM is in general multivariate (with C different channels corresponding to C MR contrasts) and has as many components as the number of classes K. The intensities are further augmented with a random Gamma transform, a standard strategy to augment generalization ability. Specifically, the GMM parameters and HR intensities are randomly sampled as follows:

(2) $$\begin{array}{cc} \mu_{k,c}∼ & N(m_{k,c}^{\mu },a_{k,c}^{\mu }),\\ \sigma_{k,c}∼ & N_{trunc}\left(m_{k,c}^{\sigma },a_{k,c}^{\sigma }\right),\\ G_{c}^{\prime }\left(x,y,z\right)∼ & N\left(\mu_{L^{T}\left(x,y,z\right),c},\sigma_{L^{T}\left(x,y,z\right),c}^{2}\right),\\ \gamma_{c}∼ & U(a_{\gamma },b_{\gamma }),\\ G_{c}= & \min_{x,y,z}G_{c}^{\prime }+\left(\max_{x,y,z}G_{c}^{\prime }-\min_{x,y,z}G_{c}^{\prime }\right)\times \\ & {\left[\frac{G_{c}^{\prime }-\min_{x,y,z}G_{c}^{\prime }}{\max_{x,y,z}G_{c}^{\prime }-\min_{x,y,z}G_{c}^{\prime }}\right]}^{\gamma_{c}},\\ G(x,y,z) & ={\left\{G_{c}\left(x,y,z\right)\right\}}_{c=1,\ldots ,C}, \end{array}$$

where the mean and standard deviation $\left(\mu_{k,c},\sigma_{k,c}\right)$ of each class k and MR contrast/channel c are independently sampled from Gaussian distributions (the latter truncated to avoid negative values), and the Gaussian intensity at HR $G_{c}$ is independently sampled at each spatial location (x,y,z) from the distribution class indexed by the corresponding label $L^{T}\left(x,y,z\right)$. Note that these Gaussians model both the variability in intensities within each label, and the actual noise in the images; modeling them simultaneously saves one step in the data generation, which is repeated at every minibatch (hence saving a non-negligible amount of time). We further assume the covariances between the different contrasts to be zero, i.e., each channel is sampled independently.

The hyperparameters $\left\{m_{k,c}^{\mu }\right\},\left\{a_{k,c}^{\mu }\right\},\left\{m_{k,c}^{\sigma }\right\},\left\{a_{k,c}^{\sigma }\right\}$ control the contrast of the synthetic images; the practical procedure we follow to estimate these parameters is detailed in Section 2.2.3 below. Finally, the parameters $a_{\gamma },b_{\gamma }$ of the uniform distribution for γ control the maximum strength of the nonlinear gamma transform. We note that this highly flexible process generates a very wide variety of contrasts – much wider than what one encounters in practice. Our goal is not to faithfully reproduce the image formation model of MRI (please see example of residual maps in Figure S1 of the supplementary material), but to generate a diverse set of images, as there is increasing evidence that exposing CNNs to a broader range of images than they will typically encounter at test time improves their generalization ability (see for instance Chaitanya et al., 2019).

#### 2.1.4 Synthetic, corrupted LR intensities

The last step of the synthetic data generation is the simulation of variability in coordinate frames and of image artifacts, including bias field, PV, registration errors, and resampling artifacts.

**Fig. 2:**
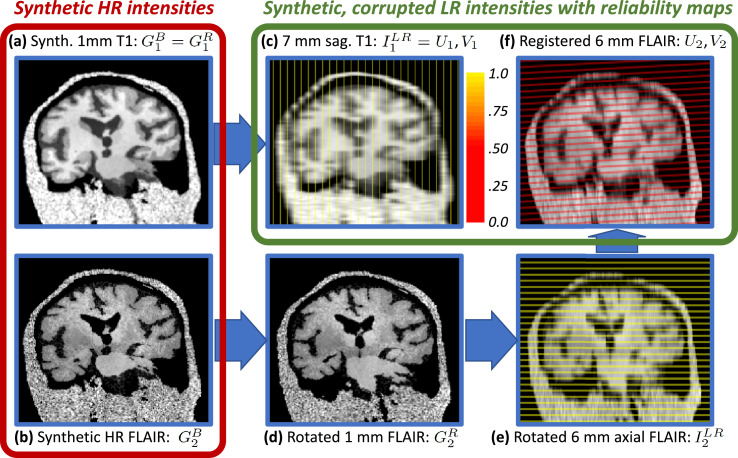
Details of the workflow for the generator of synthetic scans with reliability maps, using an example with a 7 mm sagittal T1 acquisition (used as reference) and a 6 mm axial FLAIR. (a) Synthetic HR T1 with bias field ($G_{1}^{B}=G_{1}^{R}$). (b) Synthetic HR FLAIR with bias field ($G_{2}^{B}$). (c) Synthetic LR sagittal T1 with reliability map overlaid ($I_{1}^{LR}=U_{1}$ and $V_{1}$). (d) Synthetic HR FLAIR with small random deformation, simulating subject motion in between scans ($G_{2}^{R}$). (e) Synthetic LR axial FLAIR with reliability map ($I_{2}^{LR}$). (f) LR FLAIR and reliability map registered to the reference space defined by the T1 scan ($U_{2}$ and $V_{2}$); note that the reliability map is no longer binary or parallel to the axial plane. Registration errors are modeled by adding noise to the inverse of the random rigid transform when deforming back to the reference space.

*Variability in coordinate frames* In practice, the different channels of multi-modal MRI scans are not perfectly aligned due to inter-scan motion, i.e., the fact that subject moves in between scans. Therefore, a first step when processing data from an MRI exam is to select one of the input channels to define a reference coordinate frame, and register all the other channels to it. Inter-scan motion aside, the coordinate frames of the different channels are in general not perfectly orthogonal, for two possible reasons. First, it is possible that the geometric planning of the different channels is not orthogonal by design. For example, the coronal hippocampal subfield T2 acquisition in ADNI is oriented perpendicularly to the major axis of the hippocampus, and is thus rotated with respect to the isotropic 1 mm MP-RAGE acquisition. And second, the aforementioned inter-scan motion. In order to model these differences, we apply random rigid transforms to all the MR contrasts except for the reference channel, which we assume, without loss of generality, to be the first one:

(3) $$\begin{array}{cc} & \theta_{c,x}^{R}∼U\left(a_{rot},b_{rot}\right),\;t_{c,x}∼U\left(a_{t},b_{t}\right),\\ & \theta_{c,y}^{R}∼U\left(a_{rot},b_{rot}\right),\;t_{c,y}∼U\left(a_{t},b_{t}\right),\\ & \theta_{c,z}^{R}∼U\left(a_{rot},b_{rot}\right),\;t_{c,z}∼U\left(a_{t},b_{t}\right),\\ & R_{c}=\left\{ \begin{array}{cc} \text{Id.}=\text{Rigid}(0,0,0,0,0,0), & \text{if}\;c=1\\ \text{Rigid}(\theta_{c,x}^{R},\theta_{c,y}^{R},\theta_{c,z}^{R},t_{c,x},t_{c,y},t_{c,z}), & \text{if}\;c>1 \end{array}\right.\\ & G_{c}^{R}=G_{c}\circ R_{c}, \end{array}$$

where we use the same parameters of the uniform distribution of the rotation angles as in Eq. (1), $a_{t},b_{t}$ are the extremes of the uniform distribution for the translations, Rigid(·) is a rigid transform matrix consisting of the product of three rotation and three translation matrices, $R_{c}$ is the rigid transformation matrix for channel c, and $G_{c}^{R}$ is the rigidly deformed synthetic HR volume for contrast c. An example of this deformation is shown in Fig. 2(d).

*Bias field* In order to generate a smooth multiplicative bias field, we use a strategy very similar to the one we utilized for the nonlinear deformation, and which consists of four steps that are independently repeated for each MR contrast c. First, we generate a low dimensional volume (e.g., 4×4×4) by randomly sampling a zero-mean Gaussian distribution at each location independently. Second, we linearly upsample this volume to the size of the full image $G_{c}$. Third, we take the voxel-wise exponential of the volume to obtain the bias field $B_{c}$. And fourth, we multiply each channel c of the Gaussian volume $G_{c}$ by $B_{c}$ at every spatial location:

$$\begin{array}{cc} \log B_{c}^{\prime } & ∼N_{4\times 4\times 4}\left(0,\sigma_{B}^{2}\right),\\ \log B_{c} & =\text{Upsample}(\log B_{c}^{\prime }),\\ B_{c}\left(x,y,z\right) & =\exp[\log B_{c}\left(x,y,z\right)],\\ G_{c}^{B}\left(x,y,z\right) & =G_{c}^{R}\left(x,y,z\right)B_{c}\left(x,y,z\right), \end{array}$$

where the variance $\sigma_{B}^{2}$ controls the strength of the bias field, $B_{c}\left(x,y,z\right)$ the non-negative bias field at location (x,y,z), and $G_{c}^{B}\left(x,y,z\right)$ represents the corrupted intensities of (rigidly deformed) channel c.

*Resolution: slice spacing and thickness (partial voluming)* The simulation of resolution properties happens independently for every channel, and has two aspects: slice thickness and slice spacing. In scans with thin slices, these two values are often the same; however, in scans with high spacing (e.g., over 5 mm), the slice thickness is often kept at a lower value, typically about 3–5 mm, which is a good compromise between signal-to-noise ratio (thicker is better) and crispness (thinner is better, as it introduces less blurring). Slice thickness can be simulated by blurring in the direction orthogonal to the slices. The blurring kernel is directly related to the MRI slice excitation profile, which is designed with numerical optimization methods in real acquisitions (e.g., with the Shinnar-Le Roux algorithm, Pauly et al., 1991). These optimization techniques lead to a huge variability in slice selection profiles across acquisitions and platforms, which is difficult to model accurately. Instead, we use Gaussian kernels in our simulations, with standard deviations $\sigma_{S,c}$ (dependent on direction and channel) that divide the power of the HR signal by 10 at the cut-off frequency (Billot et al., 2020b). We further multiply $\sigma_{S,c}$ by a random factor α at every minibatch, where α is sampled from a uniform distribution of predefined range (centered on 1) to mitigate the impact of the Gaussian assumption, as well as to model small deviations from the nominal slice thickness.

Once the image has been blurred, slice spacing can be easily modeled by subsampling every channel in every direction with the prescribed channel-specific spacing distances. The subsampling factor does not have to be integer; trilinear interpolation is used to compute values at non-integer coordinates. This subsampling produces synthetic, corrupted, misaligned LR intensities for every channel c. The specific processing is:

$$\begin{array}{cc} \alpha & ∼U(a_{\alpha },b_{\alpha }),\\ \sigma_{S,c} & =2\alpha \log\left(10\right)/\left(2\pi \right)r_{c}/r_{targ},\\ I_{c}^{\sigma } & =G_{c}^{R}*N\left[0,diag\left(\sigma_{S,c}\right)\right],\\ I_{c}^{LR} & =\text{Resample}\left(I_{c}^{\sigma };d_{c}\right), \end{array}$$

where $a_{\alpha },b_{\alpha }$ are the parameters of the uniform prior distribution over α; $r_{c}$ is the (possibly anisotropic) voxel size of the test scan in channel c, without considering gaps between slices; $r_{targ}$ is the (often isotropic) voxel size of the training segmentations (which defines the target resolution for SR); $I_{c}^{\sigma }$ is the blurred channel c; Resample(·) is the resampling operator; $d_{c}$ is the voxel spacing of channel c; and $I_{c}^{LR}$ are the synthetic, corrupted, misaligned LR intensities. We note that $r_{c}$, $r_{targ}$ and $\sigma_{S,c}$ are 3×1 vectors, with components for the x, y and z directions. Examples of PV modeling are shown in Fig. 2(c,e).

*Registration errors and resampling artifacts* The final step of our generator is mimicking the preprocessing that will happen at test time, where the different channels will be rigidly registered to the reference channel c=1 and trilinearly upsampled to the (typically isotropic) target resolution $r_{targ}$. At that point, all images are defined on the same voxel space, and SR and synthesis become a voxel-wise regression problem. In order to simulate the registration step, one could simply invert the rigid transform modeling the variability in coordinate frames (Eq. (3)). However, registration will always be imperfect at test time, so it is crucial to simulate registration errors in our generator. The final images produced of the generator $\left\{U_{c}\right\}$ are given by:

(4) $$\begin{array}{cc} & \epsilon_{c}∼\left\{ \begin{array}{cc} \delta (\epsilon_{c}), & c=1\\ N[0,\text{diag}\left(\sigma_{\epsilon ,\theta }^{2},\sigma_{\epsilon ,\theta }^{2},\sigma_{\epsilon ,\theta }^{2},\sigma_{\epsilon ,t}^{2},\sigma_{\epsilon ,t}^{2},\sigma_{\epsilon ,t}^{2}\right)], & c>1 \end{array}\right.\\ & R_{c}^{\prime }=R_{c}^{-1}\times \text{Rigid}\left(\epsilon_{c}\right),\\ & U_{c}^{\prime }=I_{c}^{LR}\circ R_{c}^{\prime },\\ & U_{c}=\text{Resample}\left(U_{c}^{\prime };r_{targ}\right), \end{array}$$

where δ(·) is Kronecker’s delta and $\sigma_{\epsilon ,\theta }^{2},\sigma_{\epsilon ,t}^{2}$ are the variances of the rotation and translation components of the registration error, which are assumed to be statistically independent. An example of a registered and resampled image is shown in Fig. 2(f), where the rotation has introduced noticeable resampling artifacts.

In addition to $\left\{U_{c}\right\}$, the generator also produces a second set of volumes ${\left\{V_{c}\right\}}_{c=1,\ldots ,C}$ that we call “reliability maps”, and which we use as additional inputs both during training and at test time. The reliability maps, which are similar to the “sampling masks” in Dalca et al. (2018), encode which voxels are measured vs. which are interpolated, and are a function of the trilinear resampling operation. While the CNN effectively learns the expected degree of blurring during training, providing the (deterministic) resampling pattern improves the performance of the CNN in practice. Voxels on slices of $I_{c}^{LR}$ have reliability one, whereas voxels between slices have reliability zero – see for instance Fig. 2(c,e). Reliabilities between zero and one are obtained due to linear interpolation when the target resolution is not an exact multiple of the slice spacing, or when applying the transformation $R_{c}^{\prime }$ (simulating the registration) to the maps in order to bring them into alignment with $\left\{U_{c}\right\}$, e.g., as in Fig. 2(f). We note that these maps are known for every image, and we use them as additional input at testing (Section 2.3).

### 2.2 Learning and inference

#### 2.2.1 Regression targets and loss

We train a CNN to predict the desired output Y from the inputs $\left\{U_{c},V_{c}\right\}$, i.e., the registered LR scans resampled at $r_{targ}$ and their corresponding reliability maps, which are generated on the fly during training. We consider two different modes of operation: SR alone, and joint SR and synthesis (Fig. 1). In the former case, we seek to recover the synthetic HR volume of the reference contrast $G_{1}^{B}=G_{1}^{R}$. Rather than predicting this image volume directly, it is an easier optimization problem to predict the residual instead, i.e., we seek to regress $Y=G_{1}^{B}-U_{1}$ from $\left\{U_{c},V_{c}\right\}$. This mode of operation does not require any real images for training.

In joint SR / synthesis, we instead seek to recover the real image intensities of standard contrast, typically MP-RAGE. If any of the input contrasts $c^{*}$ is similar to the target standard contrast (e.g., a T1-weighted scan acquired with a TSE sequence), we regress the residual, as in the SR case: $Y=I^{T}-U_{c^{*}}$. If not, we simply regress the target intensities directly: $Y=I^{T}$.

The CNN is trained with the Adam optimizer (Kingma and Ba, 2014), seeking to minimize the expectation of the L1 norm of the error:

$$\hat{\Omega }=\underset{\Omega }{\mathrm{argmin}}E[∥Y-\tilde{Y}\left(U_{1},V_{1},\ldots ,U_{c},V_{c};\Omega \right){∥}_{1}]$$

where Ω is the set of CNN weights (i.e., convolution coefficients and biases), and $\tilde{Y}\left(\cdot ,\Omega \right)$ is the output of the CNN when parameterized by Ω. The choice of the L1 norm as loss was motivated by the fact that it produced visually more realistic results in pilot experiments compared with the L2 norm or structural similarity (Wang et al., 2004).

We note that we do not use a validation dataset to decide when to stop training, since there is no ground truth available in our scenario. Using synthetic data generated with our model would be redundant, because these would follow the same distribution as the training data (i.e., the training and validation curves would on average be the same. Instead, we train the CNN for a fixed number of iterations (200,000), for which the loss has always converged, in practice.

#### 2.2.2 Network architecture

Our CNN builds on an architecture that we have successfully used in our previous work with synthetic MRI scans (Billot, Greve, Van Leemput, Fischl, Iglesias, Dalca, 2020, Billot, Robinson, Dalca, Iglesias, 2020). It is a 3D U-net (Çiçek, Abdulkadir, Lienkamp, Brox, Ronneberger, 2016, Ronneberger, Fischer, Brox, 2015) with 5 levels. Levels consist of two layers, each of which comprises convolutions with 3 × 3 × 3 kernels and a nonlinear ELU activation (Clevert et al., 2016). The first layer has 24 kernels (i.e., features); the number of features is doubled after each max-pooling, and halved after each upsampling. The last layer uses a linear activation to produce an estimate of Y. The U-net is concatenated with the synthetic data generator into a single model entirely implemented on the GPU, using Keras (Chollet et al., 2015) with a Tensorflow backend (Abadi et al., 2016).

#### 2.2.3 Hyperparameters

The generator described in Section 2.1 has a number of hyperparameters, which control the variability of the synthetic scans, in terms of both shape and appearance. Table 1 summarizes the values of the hyperparameters related to shape, bias field, gamma augmentation, variability in coordinate frames and slice thickness, and misregistration. These hyperparameters were set via visual inspection of the output, such that the generator yields a wide distribution of shapes, artifacts and intensity profiles during training – which increases the robustness of the CNN. Specifically, we used the same values that provided good performance in previous work (Billot, Greve, Van Leemput, Fischl, Iglesias, Dalca, 2020, Billot, Robinson, Dalca, Iglesias, 2020).

**Table 1:** Model hyperparameters. Angles are in degrees, and spatial measures are in mm.

$a_{rot}$	$b_{rot}$	$a_{sc}$	$b_{sc}$	$a_{sh}$	$b_{sh}$	$\sigma_{T}^{2}$	$a_{\gamma }$	$b_{\gamma }$	$\sigma_{B}^{2}$	$a_{b}$	$b_{t}$	$a_{\alpha }$	$b_{\alpha }$	$\sigma_{\epsilon ,\theta }^{2}$	$\sigma_{\epsilon ,t}^{2}$
10	10	log0.9	log1.1	0.01	0.01	$3^{2}$	0.7	1.3	$0.5^{2}$	20	20	0.8	1.2	$0.3^{2}$	$0.3^{2}$

The hyperparameters that control the GMM parameters $\left\{m_{k,c}^{\mu }\right\},\left\{a_{k,c}^{\mu }\right\},\left\{m_{k,c}^{\sigma }\right\},\left\{a_{k,c}^{\sigma }\right\}$ do not have predefined values, since they depend on the MR contrast – and to less extent, the resolution – of the dataset that we seek to super-resolve. For every experimental setup, we estimate them with the following procedure. First, we run our Bayesian, sequence-adaptive segmentation algorithm (SAMSEG, Puonti et al., 2016) on a small set of scans from the dataset to segment. Even though the quality of these segmentations is often low due to PV, we can still use them to compute rough estimates of the mean and variance of the intensities of each class with robust statistics. Specifically, we compute the median as an estimate for $\left\{\mu_{k,c}\right\}$, and the median absolute deviation (multiplied by 1.4826, Leys et al., 2013) as an estimate for $\left\{\sigma_{k,c}\right\}$. We then scale the estimated variances by the ratio between the volumes of the HR and LR voxels for every modality, i.e., $\left(1^{T}r_{c}\right)/\left(1^{T}r_{targ}\right)$ (where 1 is the all ones vector), such that the blurring operator yields the desired variance in the synthetic LR images. Finally, we fit a Gaussian distribution to each of the means and variances (a truncated Gaussian for the latter, in order to avoid non-negative variances) to obtain $\left\{m_{k,c}^{\mu }\right\},\left\{a_{k,c}^{\mu }\right\},\left\{m_{k,c}^{\sigma }\right\},\left\{a_{k,c}^{\sigma }\right\}$. Crucially, we multiply $\left\{a_{k,c}^{\mu }\right\}$ and $\left\{a_{k,c}^{\sigma }\right\}$ by a factor of five in order to provide the CNN with a significantly wider range of images than we expect it to see at test time, thus making it resilient to variations in acquisition (as already explained in Section 2.1.3 above), as well as for alleviating segmentation errors made by SAMSEG.

### 2.3 Inference

At testing, one simply strips the generator from the trained model, and feeds the preprocessed images to super-resolve $\left\{U_{c}\right\}$ together with the corresponding reliability maps $\left\{V_{c}\right\}$. The process to obtain these preprocessed images is the same as in Section 2.1.4 above. The first step is to resample all the scans to the target resolution $r_{targ}$, while computing the corresponding reliability maps. For the reference channel c=1, the resampled scan and its associated reliability map immediately correspond to $U_{1}$ and $V_{1}$, respectively. The other channels c>1 need to be rigidly registered; the warped resampled images and reliability maps become ${\left\{U_{c}\right\}}_{c=2,\ldots ,C}$ and ${\left\{V_{c}\right\}}_{c=2,\ldots ,C}$, respectively. In our implementation, we use an inter-modality registration tool based on mutual information and block matching (Modat et al., 2014, implemented in the NiftyReg package) to estimate the rigid alignments. The input volumes are finally padded to the closest multiple of 32 voxels in each of the three spatial dimensions (a requirement that stems from the 5 resolution levels of the U-net), and processed in one shot, i.e., without tiling; GPU memory is not a problem this context, due to the reduced memory requirements at test time, compared with training.

### 2.4 Other practical considerations

*Further blurring of synthetic HR images in training* In practice, we slightly blur the synthetic HR volumes $\left\{G_{c}^{B}\right\}$ with a Gaussian kernel with 0.5 mm standard deviation (Billot et al., 2020a); this operation introduces a small degree of spatial correlation in the images, making them look more realistic. This strategy produces slightly more visually appealing results in the purely SR mode, as these synthetic HR images are the target of the regression, but does not affect the output when jointly performing SR and synthesis.

*Normalization of image intensities* Both during training and at testing, we min-max normalize the input volumes to the interval [0,1]. In training, the normalization depends whether synthesis is being performed or not. In the purely SR mode, the target volume is normalized exactly the same way as the input, in order to keep the residual centered around zero. In the joint SR / synthesis mode, the targets are normalized by scaling the intensities such that the median intensity of the white matter is one.

*Computational burden* We randomly crop the images during training to 192×192×192 volumes, which enables training on a 16 GB GPU (the original size of the scans in the training dataset was 256 × 256×256 voxels, as detailed in Section 3.1 below). We set the learning rate to $10^{-4}$, and train the CNNs for 200,000 iterations, which was sufficient for convergence in all our experiments – there was minimal change in the loss and no perceptible difference in the outputs after approximately 100,000 - 150,000 iterations. Training takes approximately 12 days on a Tesla P100 GPU. Inference, on the other hand, takes approximately three seconds on the same GPU.

## 3 Experiments and results

This section presents three sets of experiments seeking to validate different aspects of *SynthSR*. First, we use a controlled setup with synthetically downsampled MP-RAGE scans from ADNI, in order to assess the SR ability of the method on a single volume, as a function of slice spacing. In the second experiment, we test the performance of the method in a joint SR / synthesis task, seeking to turn FLAIRs with spaced slices from ADNI into 1 mm MP-RAGEs. In the third and final experiment, we apply *SynthSR* to multimodal MRI exams from Massachusetts General Hospital (MGH), seeking to recover a 1 mm MP-RAGE from a set of different sequences with spaced slices.

### 3.1 MRI data

We used three different datasets in this study; one for training, and two for testing.

*Training dataset* The first dataset, which we used for training purposes in all experiments, consists of 39 T1-weighted MRI scans and corresponding segmentations. The scans were acquired on a 1.5 T Siemens scanner with an MP-RAGE sequence at 1 mm resolution, with the following parameters: TR = 9.7 ms, TE = 4 ms, TI = 20 ms, flip angle=$10^{\circ }$. The volume size was 256 × 256 × 256 voxels. This is the dataset that was used to build the probabilistic atlas for the segmentation routines of FreeSurfer (Fischl et al., 2002). The segmentations comprise a set of manual delineations for 36 brain MRI structures (the same as in Fischl et al., 2002), augmented with labels for extracerebral classes (skull, soft extracerebral tissue, fluid inside the eyes) automatically estimated with a GMM approach. Modeling of extracerebral tissues enables the application of our method to unpreprocessed images, i.e., without skull stripping.

*ADNI* The second dataset is a subset of 100 subjects from the Alzheimer’s Disease Neuroimaging Initiative (ADNI^3^), 50 of them diagnosed with Alzheimer’s disease (AD, aged 73.7 ± 7.3 years), and 50 elderly controls (aged 72.2 ± 7.9); 47 subject were males, and 53 females. We believe that n=100 is a sample size that is representative of many neuroimaging studies, and comparing AD with controls yields well-known volumetric effects that we seek to reproduce with scans with spaced slices. We used two different sets of images: T1 MP-RAGE scans with approximately 1 mm isotropic resolution, and axial FLAIR scans with 5 mm slice thickness and spacing. The subjects where randomly selected from ADNI3, which is a relatively modern subset of ADNI. We did not use quality control to select the subjects, but when two MP-RAGE scans were available, the best of the two was selected using visual inspection (by JEI). Even though no manual delineations are available for this dataset, we use automated segmentations of brain structures computed with FreeSurfer 7 (and their associated volumes) as a reference standard in our experiments.

*MGH* The third and final dataset consists of 50 subjects scanned at MGH (25 males, 25 females, aged 53.7 ± 18.6 years). Cases with large abnormalities, such as tumors or resection cavities, were excluded. The scans were downloaded from the MGH PACS and anonymized in accordance with an IRB-approved protocol, for which informed consent was waived. We selected a subset of four sequences that are acquired for most patients scanned at MGH over the last decade (including these 50): sagittal T1-weighted TSE (5 mm spacing, 4 mm thickness), axial T2-weighted TSE (6 mm spacing, 5 mm thickness), axial FLAIR turbo inversion recovery (6 mm spacing, 5 mm thickness), and 1.6 mm T1 spoiled gradient recalled (SPGR). We emphasize that, despite its apparently high spatial resolution, the SPGR sequence is a scout with short acquisition time (14 s), short TR/TE (3.15/1.37 ms), partial Fourier acquisition (6/8), and aggressive parallel imaging (GRAPPA with a factor of 3). These parameters lead to relatively blurry images with low contrast-to-noise ratio, which do not yield accurate measurements, e.g., when analyzed with FreeSurfer – as we show in the results below. No manual delineations are available for this dataset, and reliable automated segmentations are not available due to the lack of higher resolution companion scans.

### 3.2 Competing methods

As mentioned in Section 1.3, there are – to the best of our knowledge – no joint SR / synthesis methods available for single scans that adapt to MRI contrast, and which can thus be applied without the availability of a training dataset. In this scenario, we use SAMSEG as a competing method. Even though SAMSEG does not provide synthesis or SR, it provides segmentations for scans of any resolution and contrast, which we can use for indirect validation (e.g., ability to detect effects of disease). In the experiments with the MGH dataset, for which multiple scans of the same exam are available (including one with T1 contrast), we compare our method against Brudfors et al. (2018) – which is the only available method that we know of, that can readily super-resolve a set of volumes of arbitrary contrast into a HR scan.

In the experiments with ADNI (i.e., the first two), we also present results for a fully supervised approach using real scans during training. With the MGH dataset, this is not possible, as 1 mm MP-RAGE scans are not available. While this fully supervised approach is not a natural competitor of our method (since it requires a full HR, contrast- and resolution-specific training dataset), it enables us to assess the decrease in performance that occurs when synthetic images are used in training, instead of real scans. The fully supervised CNNs were trained on a separate set of 500 ADNI cases, and use the same architecture and augmentation schemes, as well as reliability maps.

### 3.3 Experiments

#### 3.3.1 Super-resolution of synthetically downsampled scans

Our first experiment seeks to assess the SR capabilities of *SynthSR* as a function of the resolution of the input. To do so, we artificially downsampled the MP-RAGE scans from the ADNI dataset to simulate 3, 5 and 7 mm coronal slice spacing, with 3 mm slice thickness in all cases. We then used our method to predict the residual between the HR images and the (upsampled) LR volumes, without any synthesis – such that training relies solely on synthetic data, as explained in Section 2.2.1. Examples of training pairs are shown in Figures S2, S3 and S4 in the supplementary material. A fully supervised CNN was also, trained with real 1 mm scans that are geometrically augmented and downsampled on the fly, i.e., with the same procedure as the synthetic scans.

**Fig. 3:**
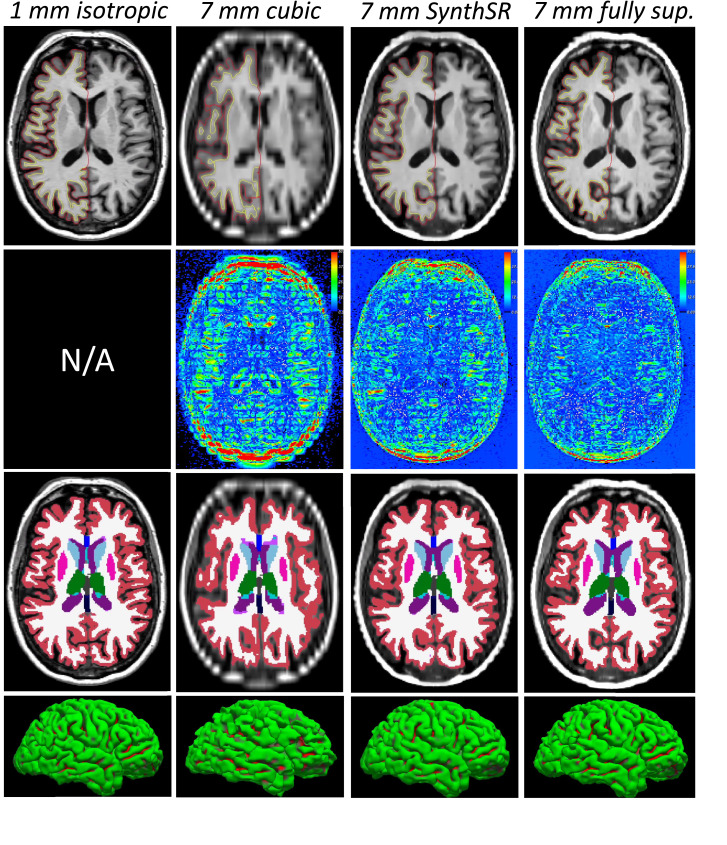
Axial slice of a sample 1 mm T1 scan from the ADNI dataset (left column); 7 mm coronal version (second column); and super-resolved back to 1 mm with *SynthSR* (third column) and the fully supervised approach (right column). Top row: image intensities with pial and white matter surfaces for the right hemisphere (computed with FreeSurfer 7). Second row: residual error maps. Third row: volumetric FreeSurfer segmentation, represented with the standard FreeSurfer color map. Bottom row: 3D rendered pial surface.

Fig. 3 shows qualitative results for a sample 7 mm scan (1 mm original, downsampled, and super-resolved with *SynthSR* and the fully supervised CNN), along with segmentations produced by FreeSurfer 7. Even though *SynthSR* has never been exposed to a real scan during training, it is able to accurately recover high-resolution features; only minimal blurring remains in the SR volume, compared with the original scan, and the residual error map is only slightly worse than that of the fully supervised CNN. When the 7 mm scan in Fig. 3 is processed directly with FreeSurfer 7 using cubic interpolation, most folding patterns are lost. However, most of these patterns are recovered when the SR volume is processed instead, both for *SynthSR* and the fully supervised approach, with almost no difference between the two. Subcortically, the segmentation of the LR scan suffers from heavy shape distortion and PV effects (e.g., peri-ventricular voxels segmented as white matter lesions, in lilac), while the *SynthSR* scan yields a segmentation almost identical to the original (and to that of the fully supervised CNN).

Table 2 shows quantitative SR results using two common metrics: peak signal-to-noise ratio (PSNR) and structural similarity index measure (SSIM, Wang et al., 2004). The former is the ratio between the maximum power of the image signal and the power of the error signal, whereas the latter uses a model seeking to mimic human perception. They were both computed with a brain mask, automatically obtained with FreeSurfer from the 1 mm isotropic scans. In terms of PSNR, *SynthSR* provides a ∼4 dB improvement with respect to cubic interpolation, and only 1–2 dB worse than the fully supervised approach, in spite of not having access to real scans. The perceptual model used by SSIM reveals a much bigger gap between cubic interpolation and our proposed technique (between 13 and 18 points), whereas the difference between *SynthSR* and the fully supervised CNN is under than 5 points at all slice spacings.

While image quality metrics like PSNR and SSIM enable direct evaluation of the SR approach, we are ultimately interested in the usability of the SR scans in downstream image analysis tasks. For this reason, we also test the performance of *SynthSR* in common neuroimaging analyses. Specifically, we asses its ability to detect differences between AD and controls in three standard tests: hippocampal volumetry, cortical thickness, and tensor-based morphometry (TBM).

**Fig. 4:**
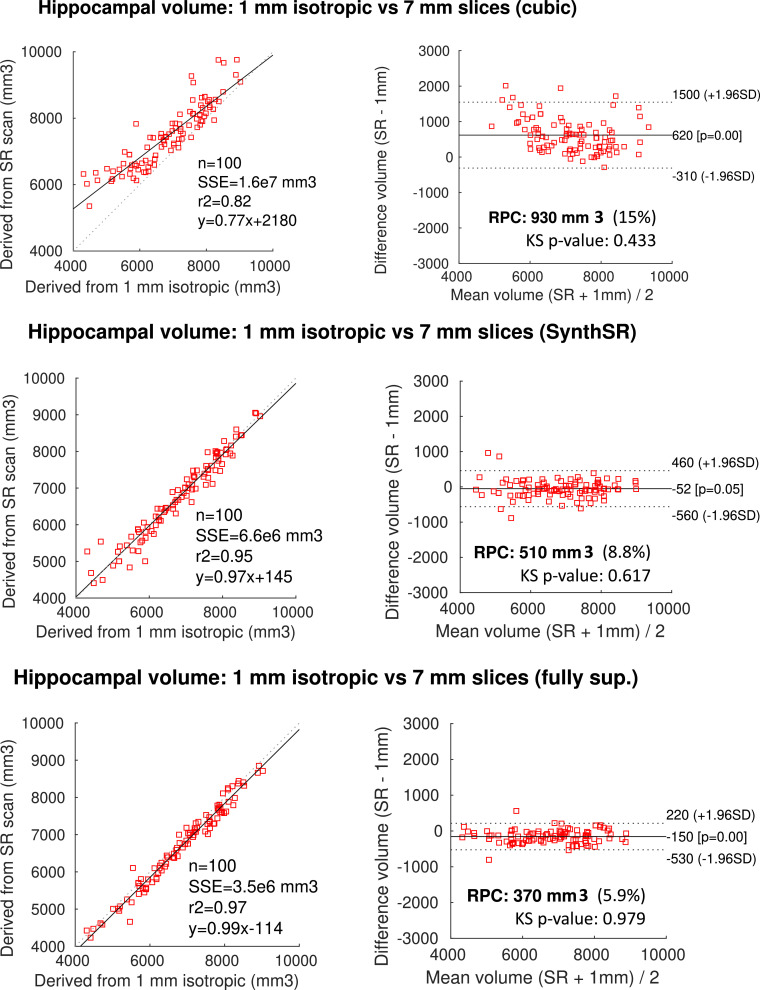
Scatter and Bland-Altman plots comparing the hippocampal volumes obtained by running FreeSurfer on the 7 mm scans vs. the ground truth, using cubic interpolation (top row), *SynthSR* (middle), and the fully supervised CNN (bottom row). In the Bland-Altmann plots, RPC stands for reproducibility coefficient, and the KS *p*-value is for a Kolmogorov-Smirnov test of normality of the differences.

*Hippocampal volumetry* Hippocampal volume is a well-known imaging biomarker for AD (Chupin, Gérardin, Cuingnet, Boutet, Lemieux, Lehéricy, Benali, Garnero, Colliot, 2009, Gosche, Mortimer, Smith, Markesbery, Snowdon, 2002, Schuff, Woerner, Boreta, Kornfield, Shaw, Trojanowski, Thompson, Jack Jr, Weiner, Initiative, 2009, Shi, Liu, Zhou, Yu, Jiang, 2009). Table 3 compares the bilateral hippocampal volume of the AD and control subjects in our ADNI dataset, using estimates of the volumes computed with FreeSurfer 7 on the 3, 5 and 7 mm scans, with and without SR. The hippocampal volumes obtained by running FreeSurfer on the 1 mm isotropic scans are used as ground truth. Without SR (i.e., just cubic interpolation), errors grow quickly with slice spacing, while SR with *SynthSR* keeps the volume errors under 3.5%, correlations between estimated and ground truth volumes over 0.97, Dice scores between the estimated and ground truth segmentations over 0.875, and effect sizes (AD vs. controls, correcting for intracranial volume, sex and age) over 1.30, even for 7 mm spacing – compared with 1.38 at 1 mm. These values are almost as small as those achieved by the fully supervised CNN (2.7% volume error, 0.99 correlation, and 0.900 Dice). The improvement with respect to the non-SR is further illustrated in the scatter and Bland–Altman plots in Fig. 4, which compares the hippocampal volumes from the 1 mm scans (i.e., the reference), with those from the 7 mm scans. Without SR, hippocampal volumes are generally overestimated, particularly for cases with lower volumes, i.e., severe hippocampal atrophy. *SynthSR*, on the other hand, consistently agrees with the reference across the whole range, and is almost as accurate has the fully supervised CNN – and interestingly, exhibits a lower bias, 52 vs. 150 mm${}^{3}$.

*Cortical thickness*

**Fig. 5:**
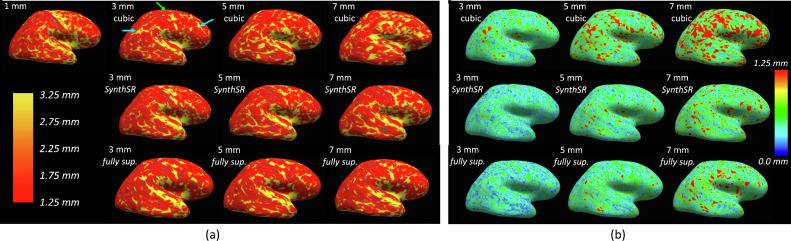
(a) Thickness map for the right hemisphere of the subject in Fig. 3, derived from different slice thicknesses, with cubic interpolation, *SynthSR*, and the fully supervised CNN. The thickness maps are displayed on the inflated surface. The blue arrows point at regions of overestimated thickness (inferior parietal, rostral middle frontal), and the green arrow points at a region where the thickness in underestimated (precentral). (b) Corresponding surface-to-surface error maps, computed as a point-wise average of the errors for the pial and white matter surfaces. (For interpretation of the references to color in this figure legend, the reader is referred to the web version of this article.)

**Fig. 6:**
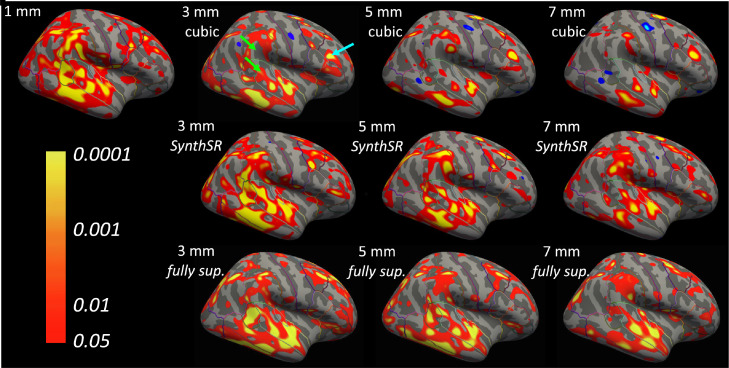
Significance maps (in logarithmic scale) for AD vs. controls in right hemisphere, corrected for age and sex, for different slice thicknesses. The top row shows the maps for cubic interpolation, the middle row for *SynthSR*, and the bottom row for the fully supervised CNN. The results are displayed on the inflated surface of FreeSurfer’s template “fsaverage”. The green arrows point at false negatives (supramarginal, superior temporal), and the blue arrow points at a false positive (rostral middle frontal). (For interpretation of the references to color in this figure legend, the reader is referred to the web version of this article.)

We conducted a similar experiment with cortical thickness, where we compared the results when analyzing 3, 5 and 7 mm coronal scans with FreeSurfer 7, and the reference obtained by running FreeSurfer 7 on the original 1 mm scans. Fig. 5 shows thickness and surface-to-surface error maps for the right hemisphere of the subject in Fig. 3; we note that we use surface-to-surface distances to visualize errors because directly comparing thicknesses would require a nonlinear registration that may be difficult, since surfaces derived from lower resolution scans miss some folds. Cortical thickness is, as expected, more sensitive to insufficient resolution than subcortical volumetry. When cubic interpolation is used, large errors appear already at 3 mm spacing, e.g., reduced thickness in precentral region, and increased thickness in inferior parietal and rostral middle frontal (see arrows in the figure). SR with *SynthSR*, on the other hand, yields a map that is very similar to the isotropic reference at 3 mm spacing; moderate errors appear at 5 mm spacing, and large errors emerge at 7 mm spacing. These errors are only very marginally higher than those incurred by the fully supervised CNN, both qualitatively (Fig. 5b) and quantitatively (Table 4). Table 4 also shows the estimated area of the pial surface: without SR, many deeper sulci are missed, leading to greatly underestimated surface areas (7.7% at 3 mm, 9.5% at 5 mm, and 13.0% at 7 mm). The SR approaches recover large part of the lost surface area, especially at 3 mm and 5 mm resolution,with *SynthSR* recovering almost as much as the fully supervised CNN. Fig. 6 shows significance maps for the AD vs. controls comparison at the group level, correcting for age and sex. The isotropic 1 mm data show expected effects in the temporal and supramarginal regions (Lehmann, Crutch, Ridgway, Ridha, Barnes, Warrington, Rossor, Fox, 2011, Lerch, Pruessner, Zijdenbos, Hampel, Teipel, Evans, 2005, Li, Wang, Wu, Shi, Zhou, Lin, Shen, Initiative, et al., 2012, Querbes, Aubry, Pariente, Lotterie, Démonet, Duret, Puel, Berry, Fort, Celsis, et al., 2009). When cubic interpolation is used, large errors render the data nearly useless already at 3 mm spacing, with false negatives in supramarginal and superior temporal regions; or false positive in rostral middle frontal; see arrows in the figure). SR with *SynthSR*, on the other hand, yields maps that are very similar to the isotropic reference at 3 mm spacing. Many clusters persist even at 5 and 7 mm, albeit with reduced significance at the group level. Very similar maps are obtained with the fully supervised CNN, showing that *SynthSR* is almost as good as using real data in this SR task.

**Table 2:** Peak signal-to-noise ratio (PSNR) and structural similarity index measure (SSIM) between the 1 mm ADNI scans and their SR counterpart – super-resolved from 3, 5 and 7 coronal scans, with cubic interpolation, *SynthSR*, and the fully supervised CNN. The metrics are computed using brain voxels only.

Slice spacing (method)	PSNR (dB)	SSIM
3 mm (cubic)	23.9 ± 0.9	0.778 ± 0.024
3 mm (*SynthSR*)	27.8 ± 1.6	0.914 ± 0.013
3 mm (fully sup.)	29.0 ± 1.4	0.938 ± 0.010
5 mm (cubic)	21.9 ± 0.9	0.688 ± 0.027
5 mm (*SynthSR*)	25.7 ± 1.5	0.854 ± 0.017
5 mm (fully sup.)	27.4 ± 1.6	0.905 ± 0.013
7 mm (cubic)	20.2 ± 1.0	0.621 ± 0.032
7 mm (*SynthSR*)	23.9 ± 1.4	0.797 ± 0.020
7 mm (fully sup.)	25.5 ± 1.5	0.842 ± 0.015

*Tensor-based morphometry* In order to assess the usefulness of the *SynthSR* volumes in registration, we investigated a TBM application (Chung, Worsley, Paus, Cherif, Collins, Giedd, Rapoport, Evans, 2001, Fox, Crum, Scahill, Stevens, Janssen, Rossor, 2001, Freeborough, Fox, 1998, Riddle, Li, Fitzpatrick, DonLevy, Dawant, Price, 2004) using a diffeomorphic registration algorithm with local normalized cross-correlation as similarity metric (Modat et al., 2010). First, we computed a nonlinear atlas in an unbiased fashion (Joshi, Davis, Jomier, Gerig, 2004, Fig. 7, top left). Then, we compared the distribution of the Jacobian determinants between AD and controls, in atlas space, with a non-parametric Wilcoxon rank sum test. The results for the different resolutions are in the same figure. The 1 mm isotropic volumes yield results that are consistent with the AD literature, e.g., contraction in the hippocampal head and tail as well as in the putamen, and expansion of ventricles (Chupin, Gérardin, Cuingnet, Boutet, Lemieux, Lehéricy, Benali, Garnero, Colliot, 2009, Hua, Leow, Parikshak, Lee, Chiang, Toga, Jack Jr, Weiner, Thompson, Initiative, et al., 2008, de Jong, van der Hiele, Veer, Houwing, Westendorp, Bollen, de Bruin, Middelkoop, van Buchem, van der Grond, 2008). Without SR (i.e., just cubic interpolation), significance already decreases noticeably at 3 mm spacing, and clusters disappear at 5 mm (e.g., hippocampal head, amygdala). Super-resolving with *SynthSR* or the fully supervised CNN, all clusters still survive at 7 mm (with minimal loss of significance strength). This indicates the power of *SynthSR* to accurately detect and quantify disease effects, even at large slice spacing, providing almost the same results as the fully supervised approach.

**Fig. 7:**
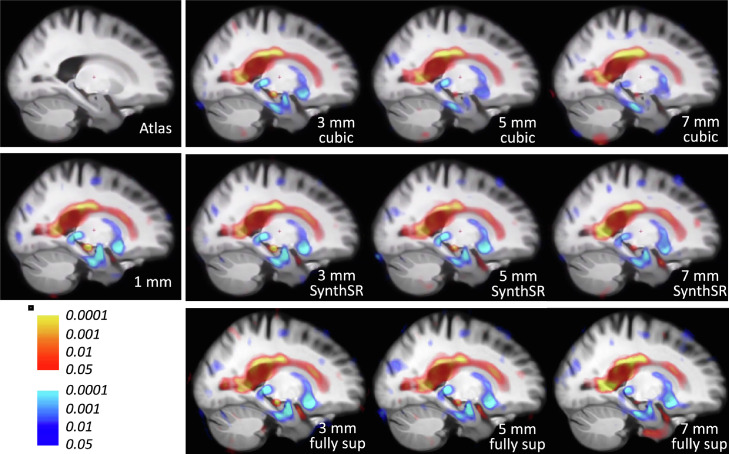
Significance maps of TBM of AD vs. controls at different resolutions, with and without SR (*SynthSR* and fully supervised CNN). Blue indicates more contraction in AD, and red indicates more expansion. (For interpretation of the references to color in this figure legend, the reader is referred to the web version of this article.)

#### 3.3.2 Joint super-resolution and synthesis of single, natively anisotropic scans

The second experiment assesses the performance the proposed method on a joint SR / synthesis problem using the FLAIR scans in ADNI. Compared with the previous experiment, where we artificially downsampled the 1 mm T1 scans, the FLAIR scans were natively acquired at 5 mm spacing (and identical thickness), with real-life slice excitation profiles. Working with ADNI scans has the advantage that we can use the measurements derived from the T1 scans as ground truth, as we did in the previous experiment. In training, we use simulated FLAIR scans as input, but, as opposed to the previous setup, we now use the real 1 mm scans as target – in order to produce synthetic scans of the reference T1 contrast, i.e., the MP-RAGE contrast of the training dataset. An example of a training pair is show in Figure S5 in the supplementary material.

**Fig. 8:**
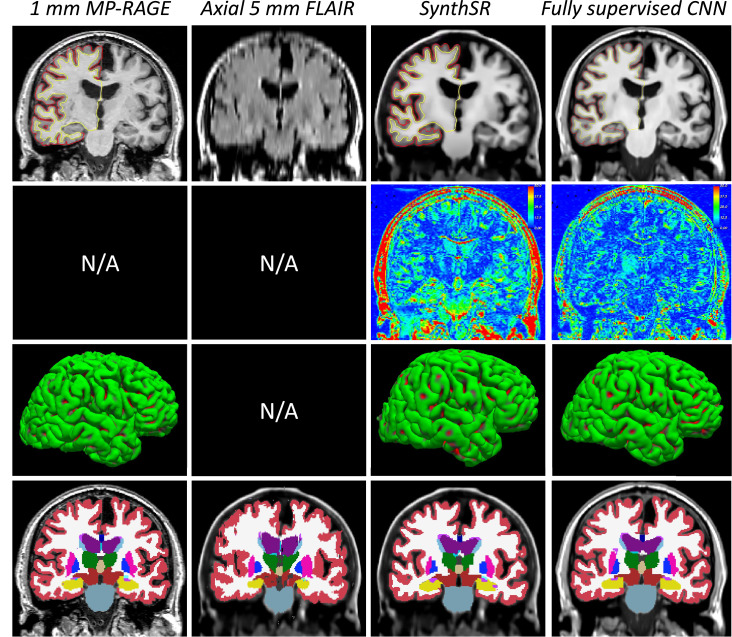
Coronal slice of a sample 1 mm T1 scan from the ADNI dataset; 5 mm axial FLAIR (with cubic interpolation); and super-resolved, with *SynthSR* and the fully supervised CNN. Top row: image intensities with pial and white matter surfaces of the right hemisphere computed with FreeSurfer 7 (not applicable to FLAIR scan). Second row: residual error maps. Third row: 3D rendering of the pial surfaces. Bottom row: volumetric segmentation obtained with FreeSurfer 7 (T1 and synthetic scans) and SAMSEG (FLAIR scan). Please note that the T1 and FLAIR scans are not perfectly aligned; we display the MP-RAGE prior to registration because resampling introduces smoothing due to interpolation artifacts (the registered scan is used to compute the residual maps).

Fig. 8 shows an example of joint SR / synthesis for one of the FLAIR scans in the ADNI dataset. The limited gray / white matter contrast of the FLAIR input makes this task much more difficult than SR of MP-RAGE scans. Nevertheless, *SynthSR* is able to recover a very good approximation of the original volume, albeit smoother than in the previous experiment (e.g., Fig. 3). While the residual maps display bigger errors that in the previous experiment, we note that this is partly due to the fact that the training and ADNI datasets have different MP-RAGE contrast (e.g., darker brainsterm, darker ventricles). This smoothness of the synthetic images leads to mistakes in the cortical segmentation, which, in spite of not appearing significant, have a large effect on cortical thickness estimation in relative terms (as shown by the results presented below), since the human cortex is only 2–3 mm thick on average. The subcortical structures, on the other hand, are a very good approximation to the ground truth obtained with the 1 mm MP-RAGE, and considerably better than the output produced by SAMSEG on the FLAIR scan upsampled with cubic interpolation, which has very visible problems – including poor cortical segmentation, largely oversegmented left putamen, or undersegmented hippocampi. Qualitatively speaking, *SynthSR* only slightly blurrier than the fully supervised CNN, and their FreeSurfer segmentations are very similar.

**Fig. 9:**
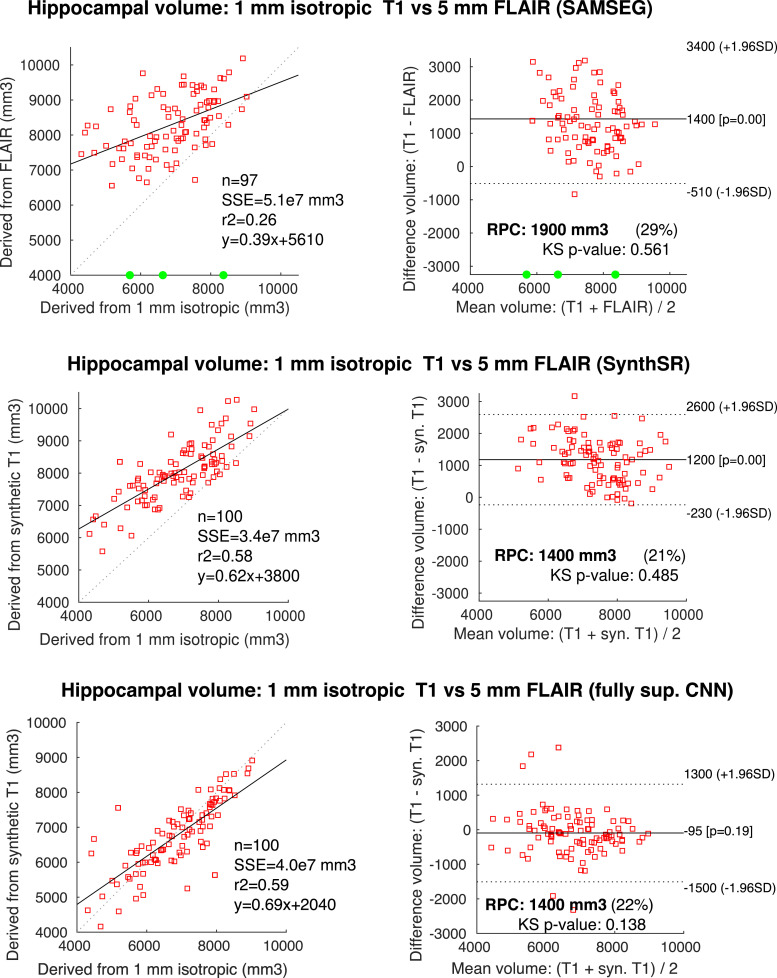
Scatter and Bland-Altman plots comparing the hippocampal volumes obtained the 1 mm MP-RAGE scans from ADNI and those from the 5 mm axial FLAIR scans, either directly (with SAMSEG, top row), or with joint SR and synthesis using FreeSurfer 7 (*SynthSR*, middle row, and fully supervised CNN, bottom row). Processing the FLAIR scans directly with SAMSEG created three outliers (in green), which were not considered in the Bland-Altman analysis. (For interpretation of the references to color in this figure legend, the reader is referred to the web version of this article.)

**Fig. 10:**
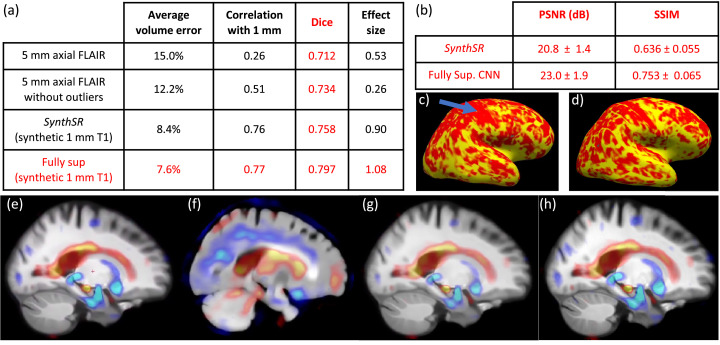
Summary of results for 5 mm axial FLAIR scans from ADNI; the ground truth is given by the measurements derived from the corresponding 1 mm MP-RAGE scans using FreeSurfer 7. (a) Relative error in hippocampal volume, correlation with volumes from 1 mm T1 scans, Dice overlap, and effect size of AD vs. controls (corrected for sex, intracranial volume and age), as in Table 3. (b) Direct image quality metrics of synthetic vs. ground truth T1 scans, as in Table 2; we emphasize that the fully supervised CNN had access to T1 scans of the target dataset (ADNI), whereas *SynthSR* did not. (c,d) Thickness maps for the right hemisphere derived from the synthesized T1 scan of the same subject as in Fig. 5, using *SynthSR* (c) and the fully supervised CNN (d); compared with the ground truth in Fig. 5 (top left), errors are rather noticeable, e.g., generally increased thickness with both approaches, and reduced thickness in the motor cortex with *SynthSR* – pointed by the arrow. (e-h) TBM using the ground truth T1 scans (e), the 5 mm FLAIR scans (overlaid on its own FLAIR atlas, f), and the synthesized MP-RAGE volumes – with *SynthSR* (g) and the fully supervised CNN (h).

Figs. 9 and 10 summarize the results for the same hippocampal volumetry, image quality metrics, cortical thickness and TBM analyses that we performed on the previous experiment. The hippocampal volumes (Fig. 9) are more spread than when doing SR alone, but are still strongly correlated with the ground truth values, particularly considering two factors: the axial acquisition (much less suitable for imaging the hippocampus than the coronal plane) and the limited contrast that the hippocampus in FLAIR. These two aspects clearly deteriorate the performance of SAMSEG, which makes much larger errors (including three outliers where the hippocampus was largely undersegmented), particularly for subjects with more severe atrophy. This is reflected in the quantitative results in Fig. 10(a): even when the outliers are disregarded, the average volume error is over 12%, the correlation is only ρ=0.51, and effect size is barely 0.26. These values greatly improve to 8.4% (volume error), ρ=0.76 (correlation) and 0.90 (effect size) respectively, when using the 1 mm T1 scans produced by *SynthSR*. Compared with the fully supervised approach, *SynthSR* provides almost the same volume error (one point higher) and correlation (one point lower), but the differences in Dice and effect size are higher (0.04 and 0.18, respectively). We note that Dice scores requires registering the FLAIR and T1 scans, and are thus affected by interpolation artifacts. This is in contrast with the estimation of volumes, or the computation of Dice scores in the previous experiments (where there was as single coordinate frame), so the results are not directly comparable.

**Table 3:** Hippocampal segmentation and volumetry using FreeSurfer on the original, downsampled (at different coronal slice spacings), and SR scans. Using the segmentations and volumes computed from the 1 mm scans as ground truth, the table reports: the average % error in hippocampal volume; the correlation with the ground truth volumes; the Dice overlap with the ground truth segmentations; and the effect size of AD vs. controls (corrected for sex, intracranial volume and age). The slice thickness was 3 mm in all cases.

Slice spacing	Average vol. error	Corr. 1 mm	Dice 1 mm	Effect size
1 mm	0.0%	1.00	1.000	1.38
3 mm (cubic)	4.5%	0.98	0.891	1.35
3 mm (*SynthSR*)	3.3%	0.99	0.901	1.36
3 mm (fully sup.)	2.7%	0.99	0.909	1.36
5 mm (cubic)	7.6%	0.95	0.863	1.22
5 mm (*SynthSR*)	2.9%	0.99	0.889	1.33
5 mm (fully sup.)	2.7%	0.99	0.904	1.35
7 mm (cubic)	10.1%	0.91	0.835	0.98
7 mm (*SynthSR*)	3.0%	0.97	0.875	1.30
7 mm (fully sup.)	2.8%	0.99	0.900	1.34

**Table 4:** Surface statistics and errors of subjects on the ADNI dataset, estimated with FreeSurfer 7 on scans of different coronal resolution, with and without super-resolution (*SynthSR* and fully supervised CNN): average surface-to-surface errors (for both pial and white matter surfaces) and average area of pial surface.

Resolution	1 mm	3 mm cubic	3 mm *SynthSR*	3 mm fully sup.	5 mm cubic	5 mm *SynthSR*	5 mm fully sup.	7 mm cubic	7 mm *SynthSR*	7 mm fully sup.
Av. pial surface-to-surface error (mm)	0.0 ± 0.0	0.51 ± 0.34	0.38 ± 0.22	0.36 ± 0.22	0.70 ± 0.50	0.49 ± 0.34	0.44 ± 0.29	0.93 ± 0.72	0.62 ± 0.48	0.56 ± 0.41
Av. white surface-to-surface error (mm)	0.0 ± 0.0	0.45 ± 0.34	0.36 ± 0.18	0.36 ± 0.22	0.66 ± 0.63	0.45 ± 0.30	0.46 ± 0.27	0.90 ± 0.86	0.55 ± 0.42	0.53 ± 0.38
Average pial surface area (cm${}^{2}$)	1,988 ± 187	1,835 ± 179	1,947 ± 185	1,989 ± 202	1,800 ± 177	1,938 ± 194	1,950 ± 192	1,729 ± 171	1,860 ± 181	1,887 ± 190

Fig. 10 (b) shows the image quality metrics (PSNR and SSIM) for the joint SR / synthesis approaches. Achieving high values for these metrics is much more difficult than in the previous experiment (SR alone), in which simple interpolation already provides a good approximation of the real intensities. The fully supervised CNN achieves metrics that are comparable to cubic interpolation of 1 × 1 × 3 m. The values for *SynthSR* are much lower, but, as mentioned above, this is largely because the method was trained to regress intensities like those of the training dataset, which have a different distribution than those in ADNI. These type of errors have little effect on downstream tasks; for example, the absolute errors in the synthesized intensities of the cerebrospinal fluid in Fig. 8 (second row) make a considerable contribution to the error (e.g., decreasing the PSNR), but do not prevent FreeSurfer from correctly segmenting, e.g., the ventricles (bottom row).

The cortical thickness maps are unfortunately not usable for this combination of contrast and resolution. Fig. 10(c,d) shows the thickness map of the subject from Fig. 8, derived with FreeSurfer 7 from the synthetic intensities provided by *SynthSR* (c) and the fully supervised CNN (d). These maps have obvious problems. For example, *SynthSR* misses the expected, highly characteristic patterns in the precentral and postcentral cortices (pointed by the arrow; please compare with the 1 mm case in Fig. 5). The fully supervised CNN also makes large errors, considerably overestimating the cortical thickness all over the hemisphere, probably due to the increased smoothness due to the SR / synthesis procedure. Registration is, on the other hand, highly successful with *SynthSR*: the TBM results (Fig. 10g) are nearly identical to those obtained with the real 1 mm T1 scans (Fig. 10e) or the fully supervised CNN (Fig. 10h), whereas using the FLAIR scans directly (with a recomputed FLAIR atlas) leads to a large number of false negatives and positives (Fig. 10f).

#### 3.3.3 Super-resolution of clinical exams with multiple scans

In this final experiment, we use the MGH dataset to evaluate *SynthSR* in the scenario it was ultimately conceived for: joint SR and synthesis on multi-modal scans with channels of different resolution and MR contrast. We use the SPGR scan as reference (i.e., register the other scans to it), and then use *SynthSR* to predict, from the four input channels, the residual between the upscaled SPGR and the desired MP-RAGE output (an example of input and target images from a minibatch is show in Figure S6 in the supplementary material). Since there is no ground truth available for this dataset, we use qualitative evaluation, as well as indirect quantitative evaluation via an aging experiment. We note that we discarded three of the 50 cases, for which FreeSurfer completely failed to segment the SPGR scan with cubic interpolation (FreeSurfer did *not* fail on the SR volume produced by our method).

**Fig. 11:**
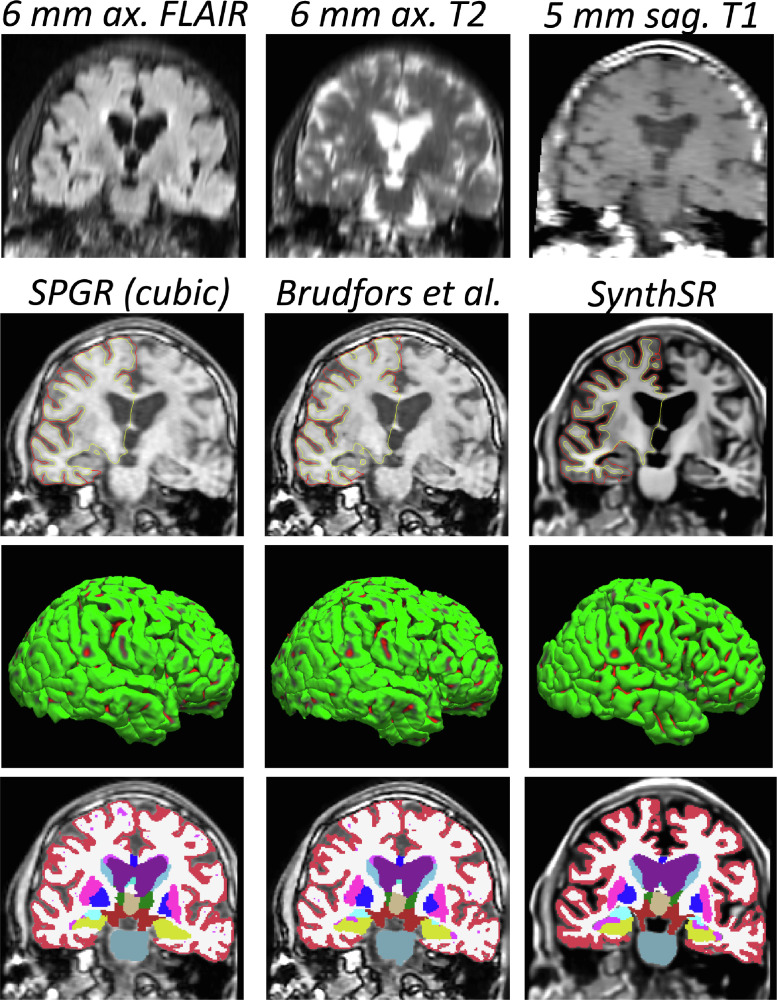
Joint SR / synthesis of an exam from the MGH dataset. The top row shows a coronal slice for the FLAIR, T2 and T1-TSE sequences, with cubic interpolation. The second row shows the corresponding T1-SPGR slice, along with the SR volume produced by Brudfors et al. (2018) and the output from our method, with the pial and white matter surfaces of the right hemisphere computed with FreeSurfer 7. The third row shows the 3D rendering of the pial surfaces. The bottom row shows the volumetric segmentation obtained with FreeSurfer 7.

**Fig. 12:**
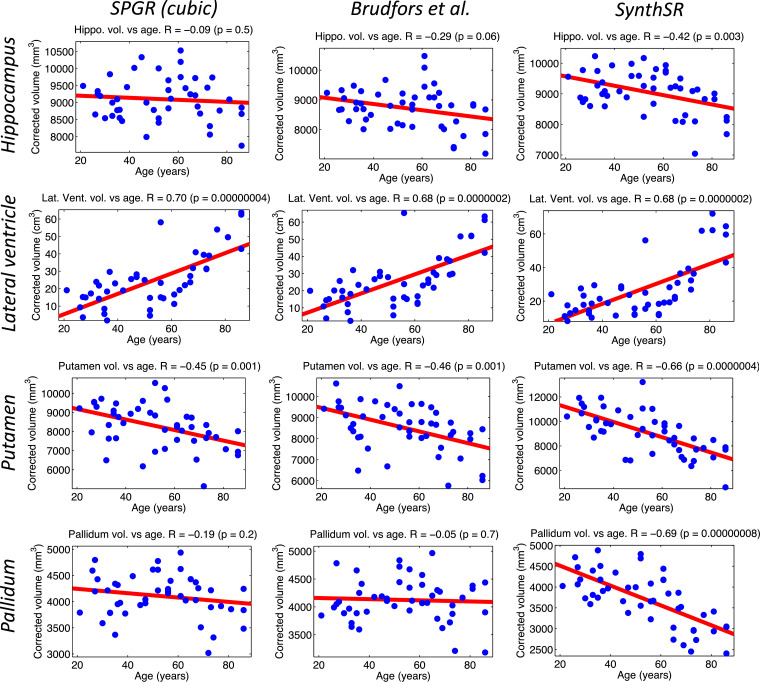
Scatter plots and linear regression of the bilateral volumes of the hippocampus, lateral ventricle and basal ganglia structures (putamen, pallidum) against age in the MGH dataset (47 subjects). The volumes were computed with FreeSurfer 7 from the SPGR scans directly (with cubic interpolation, left), their SR version produced by Brudfors et al., 2018 (middle), and the scans obtained with the joint SR / synthesis version of *SynthSR* (right). The volumes are corrected by sex and intracranial volume. The correlation coefficients and the p value for their significance are shown in the title of each plot.

Fig. 11 shows an example from the MGH dataset. Directly using the low-quality SPGR with cubic interpolation has numerous problems. Cortically, the lack of image contrast leads to poorly fitted surfaces that frequently leak into the dura matter, leading to unnaturally flat pial surfaces. Subcortically, PV and the overall lack of contrast force the FreeSurfer segmentation algorithm to heavily trust the prior; the example in the figure illustrates this problem well in the hippocampus (yellow) and the basal ganglia (putamen and pallidum, in pink and dark blue, respectively). The ability of the SPGR scans to capture well-known age effects (Potvin et al., 2016) is considerably hampered by these segmentation mistakes (Fig. 12): while very obvious large-scale features like ventricular expansion are accurately detected (even with its characteristic quadratic shape), the atrophy of the hippocampus and pallidum (correcting for sex and intracranial volume) are completely missed. Brudfors et al.’s method exploits the information on the other scans to achieve some sharpening that moderately improves the subcortical segmentation (e.g., improves the correlation of hippocampal volume and age, albeit without reaching statistical significance), while having very little effect on the placement of cortical surfaces.

Conversely, *SynthSR* yields much better contrast between gray and white matter, as well as crisper boundaries. This enhanced image quality enables FreeSurfer to generate more plausible cortical surfaces, as well as a much more precise segmentation of subcortical structures (e.g., the basal ganglia or the hippocampi in Fig. 11). This superior contrast is also reflected in the aging analysis: the volumes computed with FreeSurfer on the scans obtained with *SynthSR* successfully detect all the expected effects, i.e., atrophy of the hippocampus and basal ganglia and expansion of the lateral ventricles. The improvement with respect to Brudfors et al.’s method is very clear: *SynthSR* detects the negative slope with *p* < 0.005 for all structures, whereas their approach is completely unable to detect the slope effect in the hippocampus or pallidum, despite the fair sample size (47 subjects).

## 4 Discussion and conclusion

In this article, we have presented *SynthSR*, the first learning method that produces an isotropic volume of reference MR contrast using a set of scans from a routine clinical MRI exam consisting of anisotropic 2D acquisitions, without access to high-resolution training data for the input modalities. *SynthSR* uses random synthetic data mimicking the resolution and contrast of the scans one aims to super-resolve, to train a regression CNN that produces the desired HR intensities with the target contrast. The synthetic data are generated on the GPU on the fly with a mechanism inspired by the generative model of Bayesian segmentation, which enables simulation not only of contrast and resolution, but also changes in orientation, subject motion between scans, as bias field and registration errors. Because such artifacts and extracerebral tissue are included in the simulations, our method does not require any preprocessing, other than the rigid coregistration of the input scans (e.g., no skull stripping, denoising, or bias field correction is needed).

The first set of experiments on SR alone reveals that *SynthSR* can super-resolve MRI scans very accurately, despite the domain gap between real and synthetic data. Using artificially downsampled MP-RAGE scans from ADNI shows that one can replace 1 mm isotropic scans by super-resolved acquisitions of much lower native resolution and still detect the expected effects of disease. Our results show that, in the context of registration and subcortical segmentation, one can go down to 5 or even 7 mm slice spacing without almost any noticeable impact on common downstream analyses. Cortical thickness is, as expected, much more sensitive to larger spacing, but the proposed technique enables reliable thickness analysis at 3 mm spacing – which is remarkable, given the convoluted shape of the cortex and the small size of the thinning effect one seeks to detect.

When SR and synthesis are combined, the problem becomes much harder. Our experiments with 5 mm FLAIR scans show that cortical thickness analysis on the synthesized 1 mm MP-RAGE volumes is not reliable. Moreover, the subcortical segmentations produce volumes that yield lower effect sizes and correlations with the ground truth than when performing SR of T1 scans. However, the hippocampal volumes obtained with *SynthSR* are still usable, in absolute terms (their correlation with the ground truth volumes is over 0.75). This result is noteworthy, particularly given the axial orientation of the FLAIR scans, which is approximately parallel to the major axis of the hippocampus – causing a very robust Bayesian tool like SAMSEG to visibly falter.

The results on the MGH dataset show that *SynthSR* can effectively exploit images with different contrast and orientation. Compared with the outputs from the second experiment, the synthetic 1 mm MP-RAGEs have much better contrast in regions where it is difficult to define boundaries from a FLAIR scan alone – compare, for instance, the contrast of the putamen in Figs. 8 and 11. Even though obvious effects like ventricular expansion can be measured even with lower-resolution scans, the superior image quality produced by our approach enables FreeSurfer to reproduce subtler signatures of aging that are missed by the competing approach (e.g., pallidum). Unfortunately, as with the FLAIR scans from ADNI, the image quality of this dataset was insufficient for our method to accurately detect expected patterns of aging in cortical thickness.

We emphasize that it is not the goal of this work to replace image acquisition for a single specific subject. Rather, our goal is to enable analyses with existing neuroimaging tools that are not otherwise possible with the scans that are used in a majority of routine clinical brain MRI protocols, due to their large slice spacing. Our results show that isotropic scans synthesized with *SynthSR* can be used to compute good registrations and segmentations in many cases, almost as good as the real 1 mm scans in many analyses at the group level. Even though analysis like atrophy estimation via longitudinal segmentation or registration using the synthetic scans may be informative to evaluate a patient in clinical practice, we do not envision our method replacing specific MRI acquisitions (e.g., with contrast agents) for evaluation of abnormalities like tumors.

While it is not the goal to produce harmonized data for multi-center studies, *SynthSR* generates synthetic scans of a specific predefined MR contrast. Although this indirectly achieves a level of harmonization, it does not homogenize the data to the extent of dedicated intra-MR-contrast harmonization techniques based, e.g., on adversarial networks (i.e., trying to fool a classifier that attempts to guess the source of a scan). With *SynthSR*, the ability to generate contrast in the output depends on the quality and contrast of the input scans (e.g., as in the aforementioned example of the putamen in Figs. 8 and 11). It may thus be interesting to build a pipeline with our method and existing harmonization methods (e.g., Pomponio et al., 2020), possibly within a single architecture trained end to end.

One disadvantage of *SynthSR* is the need to train a separate CNN for every combination of orientations, resolutions, and contrasts. Even if the same training dataset can be reused, it would be preferable to be able to train a single CNN that could handle any combination of inputs, rather than having to retrain (which takes almost two weeks) every time that a new combination is encountered. Successfully training such a CNN is challenging due to the extreme heterogeneity of possible inputs and varying number of channels, but would greatly simplify deployment of *SynthSR* at scale. We will investigate this direction in future work.

Further work will also be directed towards improving the robustness and accuracy of *SynthSR*, ideally to the point that cortical thickness analyses are possible. Improving our method is possible in many aspects. In terms of loss, one could replace or complement the L1 by adversarial networks that seek to make the generated volumes indistinguishable from the training scans. While this approach generates very realistic images, it may also be prone to hallucinating image features (Cohen et al., 2018). Therefore, it will be important to compare the performance in downstream analyses. A simpler alternative may be to produce more realistic synthetic images in training by using finer labels. Crucially, labels do not need to be manual or correspond one-to-one with structures: since they are not used in learning (as opposed to, e.g., a segmentation problem), they can be obtained in an automated fashion, e.g., with unsupervised clustering techniques like Blaiotta et al. (2018).

Further improvements to *SynthSR* are also possible in terms of architecture. While the U-net in this paper combines high-level (contextual) and low-level information (finer details), and has been successfully applied to a number of related problems, it is almost certain the improved results can be obtained by tweaking the architecture. However, we the main contribution of this paper is the use of synthetic data to train CNNs for joint SR and synthesis, so a full architecture search is outside the scope of this article, and remains as future work – either by us or by others, since plugging in other architectures in our publicly available code is straightforward.

We also plan to improve the image augmentation model in the future: when deploying our method on clinical data at larger scale, the CNN will encounter images with higher degrees of noise and motion than the relatively small MGH dataset used in this study. Incorporating these artifacts into our augmentation model may improve the results. When testing at scale, we expect that some MR modalities from our minimal subset (FLAIR, T1-TSE, T2, SPGR) will be missing or unusable. While this could be addressed by training a CNN for every possible subset, we will also try training a single CNN with modality dropout. Such a CNN could potentially be applied to any MRI exam, irrespective of what modalities are available. This approach would also require the ability to automatically determine what scans within an exam are usable, which is a challenge of its own.

Finally, a key development that is required to run *SynthSR* at scale in the clinic is the ability to model pathology. The algorithm can currently only cope with atrophy (which is well modeled by spatial augmentation) and with small abnormalities, such as the moderate white matter lesions that may be encountered in ADNI. However, *SynthSR* fails to model bigger lesions that distort the brain anatomy more severely, such as tumors or stroke. One possible way of tackling this problem is to simulate such lesions during training, which could be quite difficult, depending on the spectrum of pathologies than one wishes to cover. Moreover, and given that *SynthSR* seems to be able to cope with a fair amount of domain gap between synthetic and real intensities, it is unclear how accurate these simulations will have to be. In this context, it will also be crucial to quantify uncertainty in the synthesis, and analyze how such uncertainty propagates to downstream measures. This is a challenging endeavor, since the uncertainty propagates differently through different MR contrasts and analyses (e.g., segmentation vs. registration, FSL vs. FreeSurfer), and also due to the difficulties associated with obtaining ground truth (as discussed in Section 1.2). The ability to quantify uncertainty will be particularly important when pathology is present, and models are more likely to generalize poorly.

*SynthSR* is publicly available (at https://github.com/BBillot/SynthSR) and will enable researchers around the globe to generate synthetic 1 mm scans from vast amounts of brain MRI data that already exist and are continuously being acquired. These synthetic scans will enable the application of many existing neuroimaging tools designed for research-grade MRI (including but not limited to the ones in this paper) to huge sample sizes, and thus hold promise to improve our understanding of the human brain by providing levels of statistical power that are currently not attainable with research studies.

## CRediT authorship contribution statement

**Juan Eugenio Iglesias:** Conceptualization, Methodology, Software, Formal analysis, Writing - original draft. **Benjamin Billot:** Methodology, Software, Writing - review & editing. **Yaël Balbastre:** Methodology, Software, Writing - review & editing. **Azadeh Tabari:** Validation, Resources, Data curation, Writing - review & editing. **John Conklin:** Validation, Resources, Data curation, Writing - review & editing. **R. Gilberto González:** Conceptualization, Validation, Resources, Writing - review & editing. **Daniel C. Alexander:** Methodology, Writing - review & editing. **Polina Golland:** Methodology, Writing - review & editing. **Brian L. Edlow:** Conceptualization, Writing - review & editing. **Bruce Fischl:** Conceptualization, Methodology, Writing - review & editing.

## Appendix A Supplementary materials

Supplementary Data S1 Supplementary Raw Research Data. This is open data under the CC BY license http://creativecommons.org/licenses/by/4.0/ Supplementary Data S1

Supplementary Data S2 Supplementary Raw Research Data. This is open data under the CC BY license http://creativecommons.org/licenses/by/4.0/ Supplementary Data S2

## Footnotes

2 In this article, we use the term “exam” to refer to the set of scans acquired from a subject during a single MRI session.

3 The ADNI was launched in 2003 by the National Institute on Aging, the National Institute of Biomedical Imaging and Bioengineering, the Food and Drug Administration, private pharmaceutical companies and non-profit organizations, as a $60 million, 5-year public-private partnership. The main goal of ADNI is to test whether MRI, positron emission tomography (PET), other biological markers, and clinical and neuropsychological assessment can be combined to analyze the progression of mild cognitive impairment (MCI) and early AD. Markers of early AD progression can aid in the development of new treatments and monitor their effectiveness, as well as decrease the time and cost of clinical trials. The Principal Investigator of this initiative is Michael W. Weiner, MD, VA Medical Center and University of California - San Francisco. ADNI has been followed by ADNI-GO and ADNI-2. These three protocols have recruited over 1500 adults (ages 55–90) from over 50 sites across the U.S. and Canada to participate in the study, consisting of cognitively normal older individuals, people with early or late MCI, and people with early AD. Subjects originally recruited for ADNI-1 and ADNI-GO had the option to be followed in ADNI-2.
