# Supplementary material for: Joint super-resolution and synthesis of 1 mm isotropic MP-RAGE volumes from clinical MRI exams with scans of different orientation, resolution and contrast
Source: Neuroimage. 2021 Aug 15;237:118206. doi: 10.1016/j.neuroimage.2021.118206 (PMC8354427; doi:10.1016/j.neuroimage.2021.118206)
Supplement: Supplementary Data S1 — Supplementary Raw Research Data. This is open data under the CC BY license http://creativecommons.org/licenses/by/4.0/ [file mmc1.pdf]

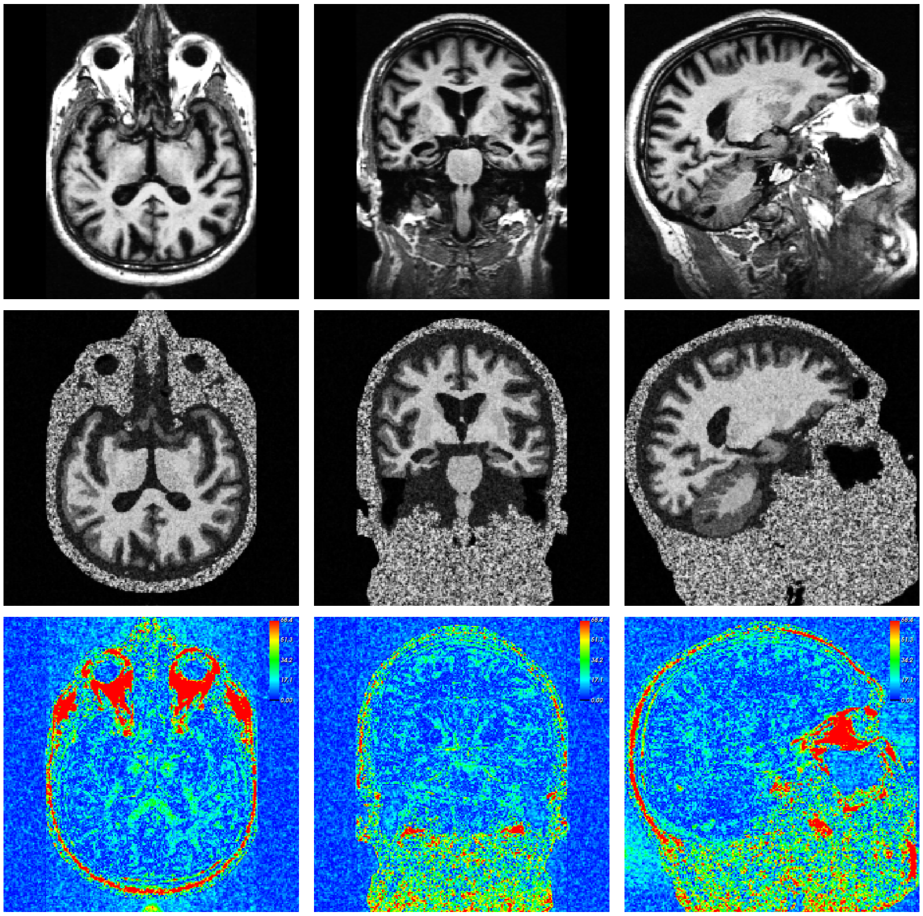

Fig. S1: Sample of Gaussian mixture model vs real scan. Top row: three orthogonal slices of a real MP-RAGE scan from the training dataset. Middle row: sample of Gaussian mixture model conditioned on the segmentation of the scan (with matched Gaussian parameters), minimally blurred for cosmetic purposes *(Billot et al., 2020a)*. Bottom row: corresponding residual maps. The biggest errors occur outside the brain, since the model for the extracerebral tissue is simplistic. Intracranially, most of the errors are high-frequency, since the Gaussian mixture is an accurate model of brain tissue.

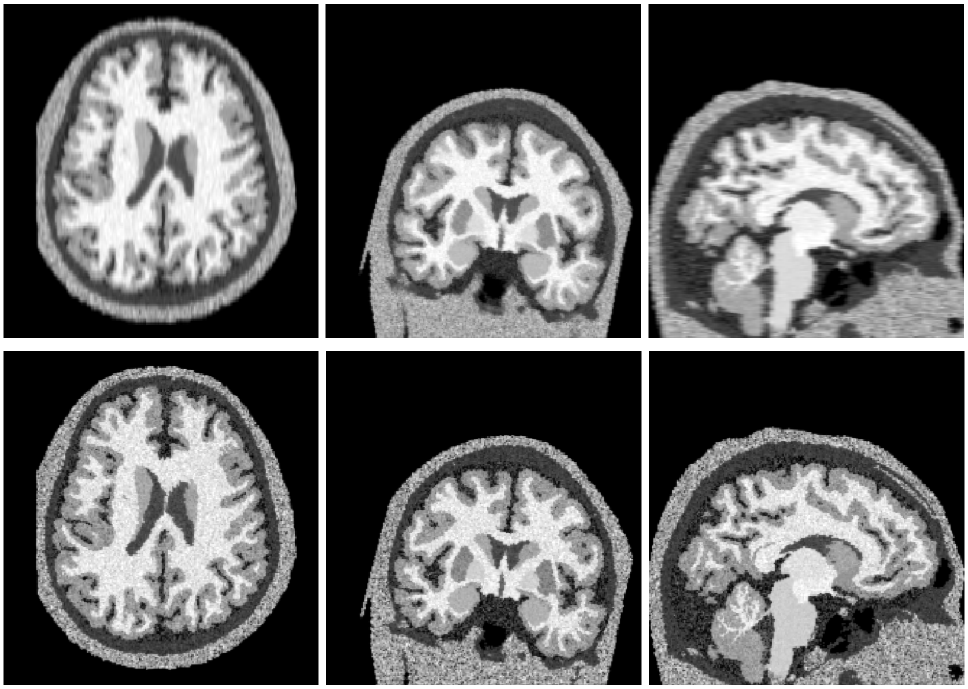

Fig. S2: Example of synthetic images used to train the 3 mm super-resolution model in the first experiment. Top row: axial, coronal and sagittal planes of a synthetic 1x1x3 mm scan, used as input to the neural network during training. Bottom row: corresponding planes on the synthetic 1x1x1 mm scan, used as target during training.

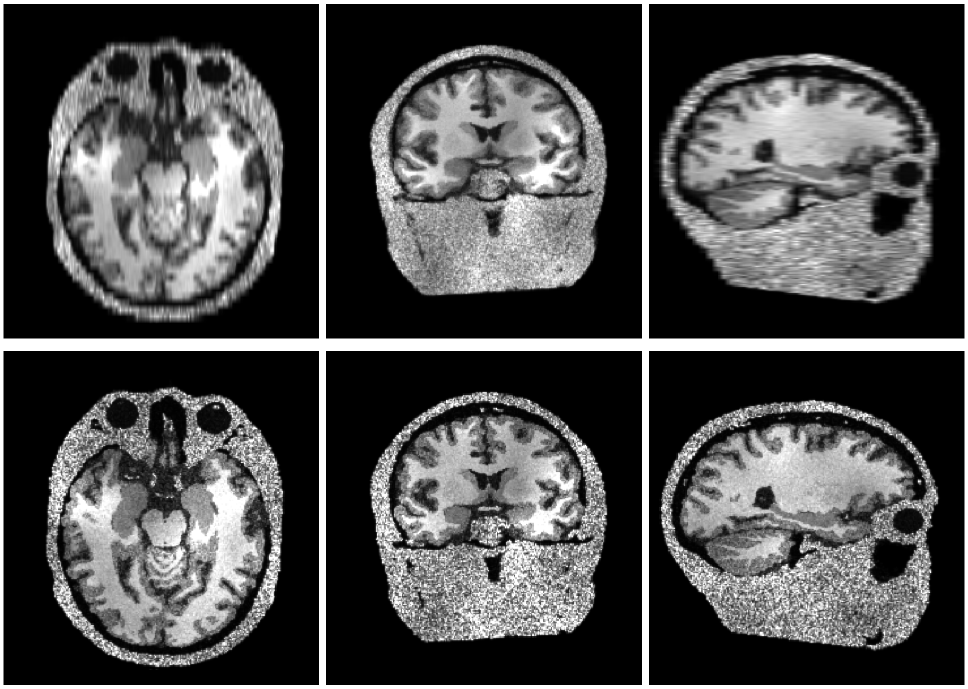

Fig. S3: Example of synthetic images used to train the 5 mm super-resolution model in the first experiment. Please see caption of Figure S1 for an explanation of this figure.

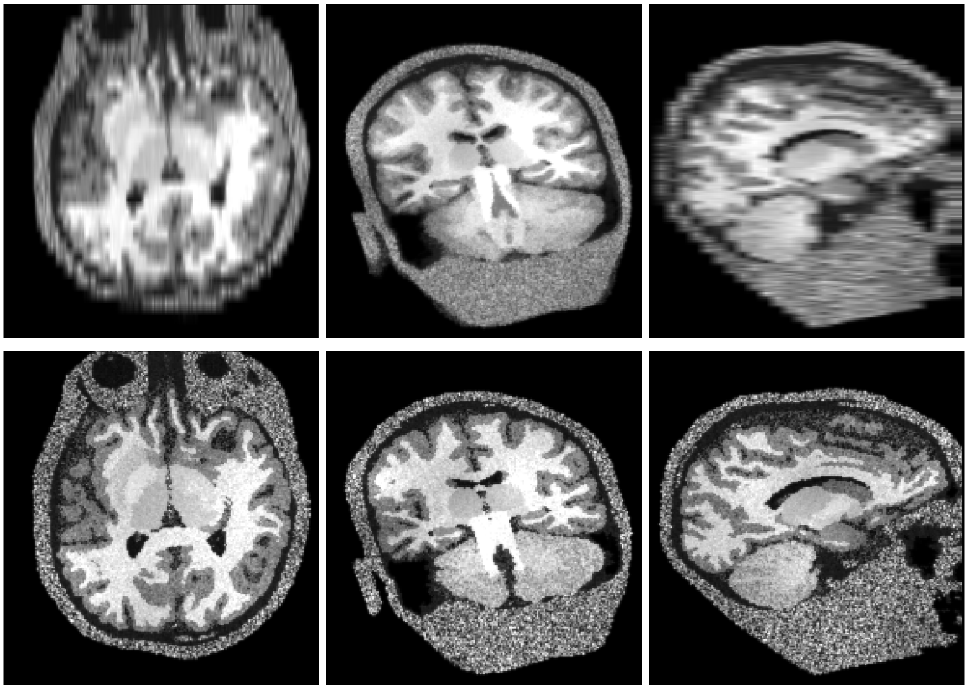

Fig S4: Example of synthetic images used to train the 7 mm super-resolution model in the first experiment. Please see caption of Figure S1 for explanation of this figure.

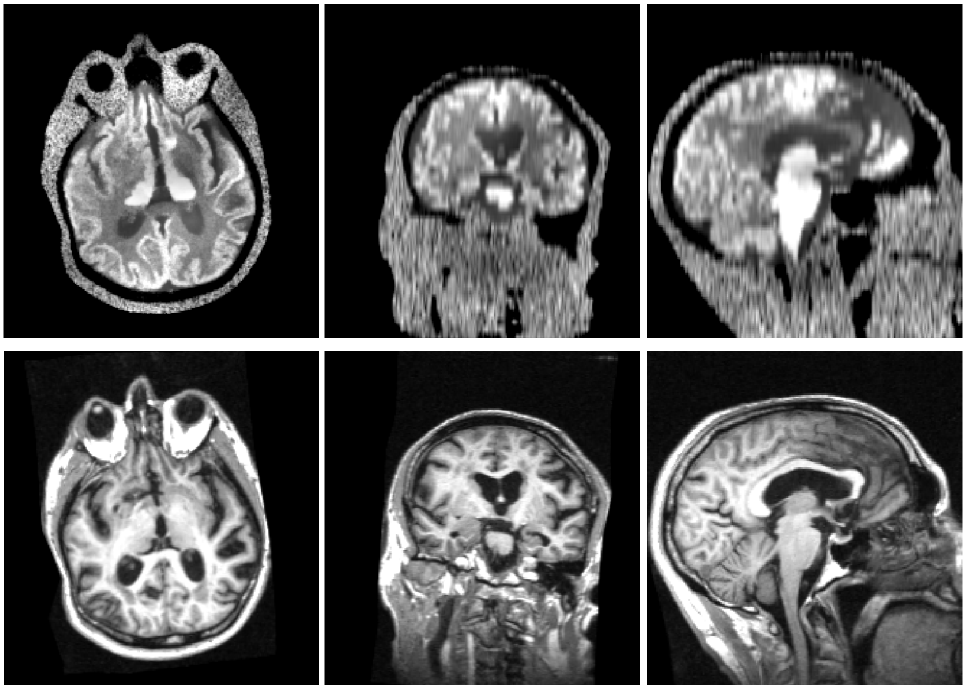

Fig. S5: Example of images used to train the joint synthesis / super-resolution model going from 5 mm axial FLAIR to 1 mm isotropic MP-RAGE. Top row: synthetic 1x1x5 mm FLAIR. Bottom row: real 1x1x1 mm MP-RAGE. We train a neural network that uses the former to estimate the latter.

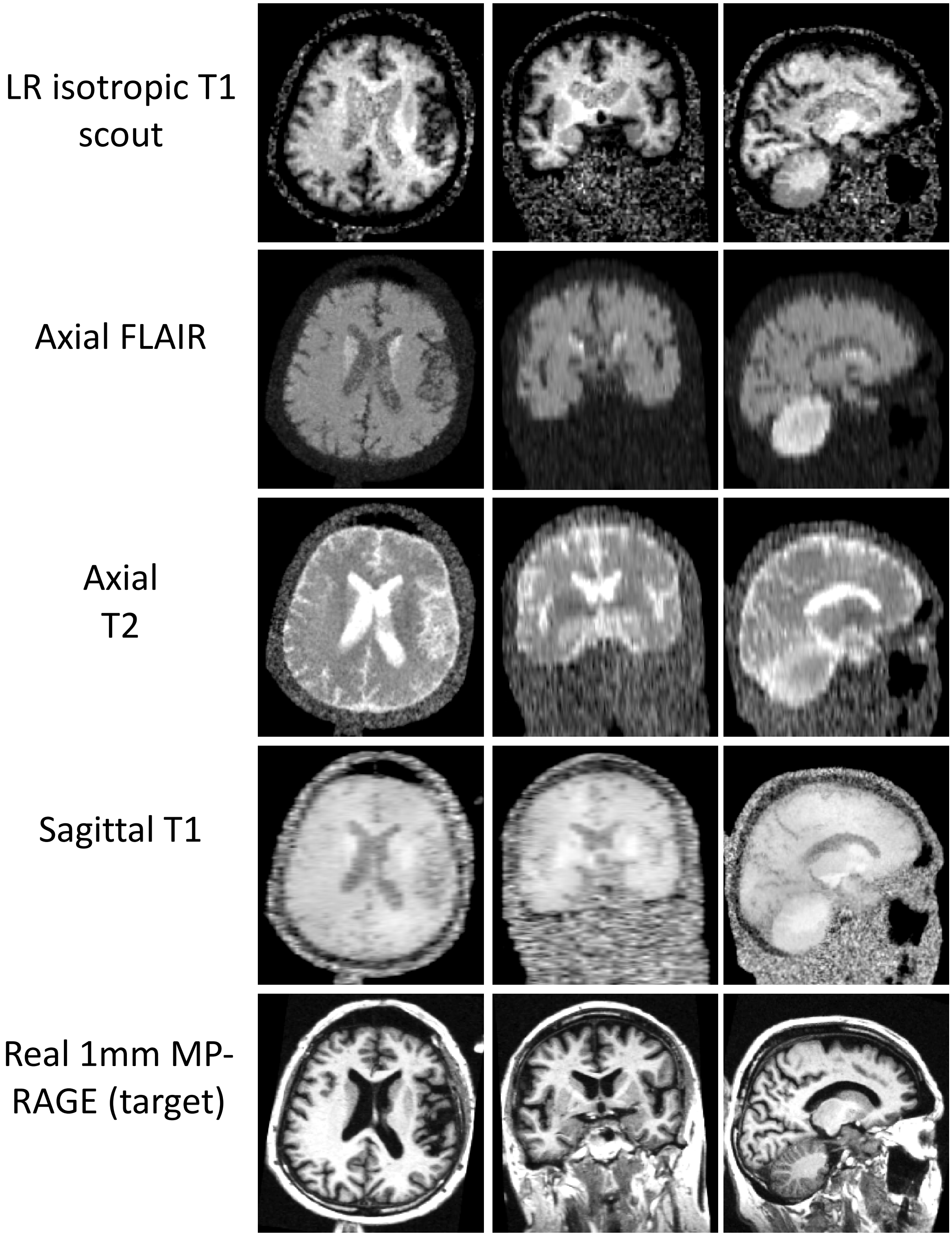

Fig. S6: Example of images used to train the joint synthesis / super-resolution model used with the clinical MGH datasets. The first four rows show the different simulated scans used as inputs, whereas the bottom row shows the real 1mm MP-RAGE used as target.
